# Screening and identification of novel anti-inflammatory peptides from sea cucumber gonads: In silico and in vitro analysis

**DOI:** 10.1016/j.fochx.2026.103510

**Published:** 2026-01-16

**Authors:** Zhiqin Zhang, Yongke Deng, Jingxuan Wang, Peipei Dou, Hongbing Fan, Xiangquan Zeng, Xinguang Fan, Haimei Liu, Qin Zhao

**Affiliations:** a College of Food Engineering, Ludong University, Yantai, Shandong 264025, PR China; b Department of Animal and Food Sciences, University of Kentucky, Lexington, KY, 40546, USA; c Department of Food Science, College of Agriculture, Purdue University, West Lafayette, IN, United States

**Keywords:** Sea cucumber gonad, Anti-inflammatory peptides, Lipopolysaccharide, Pro-inflammatory cytokines, Molecular docking

## Abstract

This study identified three novel anti-inflammatory peptides from sea cucumber gonad hydrolysates: GDRGF, FDGPEGPRGPPGSEGRQG, and PSNLGTGLR. In vitro experiments demonstrated that these peptides, at concentrations ranging from 25 to 400 μg/mL, exhibited no cytotoxicity toward RAW264.7 macrophages and significantly promoted cell proliferation (*P* < 0.05). In an lipopolysaccharide (LPS)-induced inflammation model, all three peptides markedly suppressed the secretion of nitric oxide (NO) and pro-inflammatory cytokines (*P* < 0.05), with GDRGF exhibiting the highest NO inhibition rate of 62.89%. Molecular docking results confirmed that the three peptides interact with Toll-like receptors 2 and 4 receptors via hydrogen bonding and hydrophobic interactions. Analysis of key residues suggested that internal or C-terminal arginine (R) may enhance anti-inflammatory activity by inhibiting NO production and blocking LPS signaling pathways. This study provides an efficient screening strategy for marine-derived anti-inflammatory peptides and lays a theoretical foundation for developing anti-inflammatory functional foods from sea cucumber gonads.

## Highlights

• Three anti-inflammatory sea cucumber gonad peptides were noncytotoxic and promoted RAW264.7 proliferation at 25-400 μg/mL.
• They significantly inhibited LPS-induced NO and pro-inflammatory cytokines (TNF-α, IL-1β, IL-6).
• Molecular docking revealed high-affinity binding (≤ −7.0 kcal/mol) via hydrogen bonds and hydrophobic interactions.
• Internal and C-terminal arginine residues may block LPS signaling and suppress NO production.

## 1 Introduction

Inflammation is a highly conserved physiological and pathological response triggered by the body to combat infection, tissue damage, or harmful stimuli (Yang et al., 2025b). Its core features include immune cell activation, release of inflammatory mediators, and vascular responses (Zhao et al., 2024). However, when inflammatory regulation becomes imbalanced, excessive or persistent inflammatory reactions can lead to tissue damage (Luo et al., 2024) and contribute to the pathogenesis of various chronic diseases, such as rheumatoid arthritis, atherosclerosis, neurodegenerative disorders, and cancer (Jomova et al., 2025). Currently, clinical anti-inflammatory treatments primarily rely on non-steroidal anti-inflammatory drugs (NSAIDs) and corticosteroids (Shafiq et al., 2025). However, long-term use of these drugs may induce gastrointestinal complications, including dyspepsia, nausea, vomiting, abdominal pain, and heartburn (Sostres et al., 2010), increase the risk of cardiovascular diseases (Chapa-Villarreal et al., 2024), and incur high treatment costs. Therefore, the development of novel anti-inflammatory molecules with high efficacy and low toxicity has become a research priority. In recent years, naturally derived bioactive peptides have demonstrated significant potential due to their high safety profile and favorable membrane permeability.

Toll-like receptor 2 (TLR2) and Toll-like receptor 4 (TLR4), as crucial pattern recognition receptors in the innate immune system (Park et al., 2023), play a vital role in recognizing pathogen-associated molecular patterns (PAMPs) and damage-associated molecular patterns (DAMPs). They are primarily expressed on the surface of immune cells such as macrophages and dendritic cells, where they recognize components like lipopolysaccharide (LPS), lipoproteins, and peptidoglycans, thereby initiating innate immune responses (Cutolo et al., 2022). Upon activation, TLR2 and TLR4 trigger the myeloid differentiation primary response 88 (MyD88)-dependent signaling pathway, ultimately leading to the activation of nuclear factor-κB (NF-κB) and mitogen-activated protein kinase (MAPK) signaling pathways, which promote the production of key pro-inflammatory cytokines such as interleukin-1β (IL-1β), interleukin-6 (IL-6), and tumor necrosis factor-α (TNF-α) (Lopez et al., 2024). Specifically, TLR4 forms a complex with the accessory protein myeloid differentiation protein 2 (MD2) to recognize LPS from Gram-negative bacteria, whereas TLR2 primarily detects peptidoglycans and lipoproteins from Gram-positive bacteria (Zhao, Pang, Gao, Li and Jiang, 2025). However, excessive activation of Toll-like receptor (TLR) signaling pathways is closely associated with various inflammation-related diseases, including rheumatoid arthritis and inflammatory bowel disease (Wayal et al., 2025), making them important targets for anti-inflammatory therapy.

Natural anti-inflammatory agents have long held significant importance in traditional medicine and modern nutrition. Phytochemicals (e.g., flavonoids and polyphenols) (Chen et al., 2025) and traditional Chinese herbs (e.g., turmeric and honeysuckle) (Jain et al., 2025) have been widely utilized due to their notable anti-inflammatory properties. In recent years, with advancements in biotechnology, peptide-based substances have emerged as a new research focus in anti-inflammatory studies owing to their unique bioactivity and low toxicity.

Anti-inflammatory peptides, as a novel class of immunomodulators, exhibit great potential in regulating TLR signaling pathways due to their low toxicity, high specificity, and multi-target effects. Research have revealed that anti-inflammatory peptides from various sources can bind to TLR2/4, suppressing their signal transduction and thereby alleviating inflammatory responses. For instance, casein-derived peptides ALPMHIR, AMKPWIQPK, and NPWDQVKR can form strong interactions with TLR2/4 to inhibit inflammation (Jiang et al., 2024a). The anti-inflammatory peptide KEKKEVVEYGPSSYGYG competitively binds to the hydrophobic pocket of MD2, blocking the interaction between LPS and the TLR4-MD2 complex and consequently suppressing downstream inflammatory signaling pathways (Wang et al., 2024a). Furthermore, anti-inflammatory peptides can modulate key signaling pathways to exert their effects. For example, silkworm pupa-derived anti-inflammatory peptides inhibit IκB kinase (IKK) activity, prevent inhibitor of NF-κB (IκB) degradation, block NF-κB nuclear translocation, and suppress phosphorylation of the MAPK family (p38, c-Jun N-terminal kinase (JNK), extracellular signal-regulated kinase (ERK)), ultimately reducing the production of pro-inflammatory cytokines (Zhou et al., 2024).

Virtual screening and molecular docking techniques, as core methodologies in computer-aided drug design (CADD), play a pivotal role in the systematic identification and optimization of lead compounds. In the development of antihypertensive and antidiabetic drugs, virtual screening has been successfully applied to systematically identify inhibitors targeting angiotensin-converting enzyme (ACE) and dipeptidyl peptidase-IV (DPP-IV), significantly improving the efficiency of target-specific molecule discovery while reducing research costs (Liang et al., 2025). Molecular docking enables the prediction of interaction patterns between anti-inflammatory peptides and TLR2/4 receptors. This approach not only facilitates the screening of peptide sequences with high affinity and specificity but also provides atomic-level insights into peptide-receptor binding mechanisms. Recent studies demonstrated the successful identification of Hit12 from an initial library of 257,706 compounds through virtual screening and molecular docking. This compound effectively suppresses LPS-induced TLR4/MD2 complex formation, thereby blocking the activation of NF-κB and MAPK signaling pathways (Yang et al., 2025a). These findings not only offer novel strategies for structural optimization in developing innovative anti-inflammatory agents but also establish a systematic molecular theoretical framework for the rational design of functional food bioactive components by elucidating precise peptide-receptor interaction networks. This highlights the broad interdisciplinary application prospects of CADD technologies in cutting-edge research.

The sea cucumber gonad, serving as the reproductive organ of sea cucumbers, is one of the primary by-products in sea cucumber processing. It is rich in proteins, amino acids, and unsaturated fatty acids (Sumarto & Karnila, 2022). For many years, the body wall of sea cucumbers has been the main raw material for the development of bioactive peptides in the sea cucumber industry(Senadheera et al., 2023; Shou et al., 2023). However, with the advancement of deep-processing technologies and the high added value of sea cucumber gonad, the biological activities of peptides derived from sea cucumber gonad-such as antioxidant, anti-fatigue, and ACE inhibitory effects-have gradually become research hotspots in the sea cucumber industry (Li, Gao, et al., 2025; Lu et al., 2022; Ye et al., 2017). In contrast, studies on their anti-inflammatory activity remain relatively limited, and the structural characteristics and mechanisms of action of anti-inflammatory peptides from sea cucumber gonad require systematic and in-depth investigation.

This study aimed to identify novel anti-inflammatory peptides from sea cucumber gonad hydrolysates using liquid chromatography-tandem mass spectrometry (LC-MS/MS) and computer-aided simulation techniques. The anti-inflammatory activity of the screened peptides was validated using an LPS-induced RAW264.7 macrophage inflammation model. Furthermore, molecular docking was employed to elucidate the potential binding mechanisms between the anti-inflammatory peptides and TLR2/4. The research seeks to achieve efficient screening and mechanistic investigation of sea cucumber gonad-derived anti-inflammatory peptides, thereby promoting the high-value utilization of this protein resource and providing a crucial theoretical foundation for developing marine-derived functional anti-inflammatory peptides.

## 2 Materials and methods

### 2.1 Materials and chemicals

All chemicals and reagents were obtained from commercial suppliers in China. Pepsin was purchased from Sangon Biotech (Shanghai) Co., Ltd. (China). Flavourzyme was obtained from Solarbio Science & Technology Co., Ltd. (Beijing, China). DMEM high-glucose medium, penicillin-streptomycin solution, fetal bovine serum (FBS), and phosphate-buffered saline (PBS) were acquired from Procell Life Science & Technology Co., Ltd. (Wuhan, China). The CCK-8 cell viability assay kit was sourced from Elabscience Biotechnology Co., Ltd. (Wuhan, China). The NO assay kit was purchased from Beyotime Biotechnology (Shanghai, China). LPS and ELISA kits (for TNF-α, IL-6 and IL-1β detection) were obtained from Solarbio Science & Technology Co., Ltd. (Beijing, China).

### 2.2 Preparation and isolation of enzymatic hydrolysates from sea cucumber gonads

The sea cucumber gonad tissues were homogenized and subjected to a two-stage defatting process using isopropanol at a 1:4 (*w*/*v*) ratio. The first defatting was conducted at 35 ± 2 °C for 1 h, followed by a second defatting under identical conditions for 90 min (Wang et al., 2013). The defatted samples were then mixed with deionized water at a 1:5 (g/mL) ratio for enzymatic hydrolysis. Sequential enzymatic digestion was performed, first with pepsin (2000 U/g, pH 2.5) at 37 °C for 4 h, followed by flavourzyme (1200 U/g, pH 7.0) at 50 °C for 2 h. The enzymatic reactions were terminated by boiling at 100 °C for 10 min, and the supernatant was collected after centrifugation at 5000 r/min for 15 min. The resulting hydrolysate was fractionated using spiral-wound membranes with molecular weight cut-offs of 200 Da and 3000 Da to obtain peptide fractions ranging from 200 to 3000 Da. The peptide fractions were freeze-dried to obtain peptide powder, which was stored at −18 °C for subsequent experiments.

### 2.3 Determination of molecular weight distribution of peptide fractions

The method was optimized based on reference (Xun et al., 2025) using a TSK-GEL G2000SWXL column (7.8 × 300 mm) with an isocratic mobile phase consisting of acetonitrile-water-trifluoroacetic acid (20:80:0.1, *v*/v/v) at a flow rate of 0.5 mL/min. Detection was performed at 220 nm with the column maintained at 25 °C*. prior* to analysis, samples (10 μL injection volume) were filtered through a 0.22 μm membrane. Calibration curve was established for molecular weight determination using cytochrome C (12,365 Da), aprotinin (6511 Da), bacitracin (1450 Da), oxidized glutathione (612 Da), reduced glutathione (307 Da), and L-tryptophan (204 Da) as standards. The resulting log (MW)-retention time relationship followed the linear equation Y = −0.199× + 6.5188 (R^2^ = 0.9976), which was subsequently applied to determine the molecular weight distribution of enzymatic hydrolysates.

### 2.4 Peptide sequence identification and virtual screening

#### 2.4.1 Identification and analysis by liquid chromatography-tandem mass spectrometry

The samples were mixed with 0.1% trifluoroacetic acid (TFA) and centrifuged at 20,000 *g* for 5 min. The supernatant was transferred to a 10 kDa ultrafiltration tube and centrifuged at 12,000 *g* for 15 min. After repeating the addition of 200 μL 0.1% TFA and centrifugation twice, the filtrate was collected, desalted using C18 StageTip, and vacuum dried. The dried peptide fraction was reconstituted in 0.1% formic acid (FA) for concentration determination prior to LC-MS/MS analysis. Peptide separation was performed using a nano-flow Easy nLC 1200 chromatography system with mobile phase A (0.1% FA in water) and B (80% acetonitrile containing 0.1% FA) (Mustikasari et al., 2024). The column was equilibrated with mobile phase A, and samples were pre-separated on a trap column before analytical column separation at a flow rate of 300 nL/min. The gradient program was as follows: 2–5% B (0–2 min), 5–28% B (2–44 min), 28–40% B (44–51 min), 40–100% B (51–53 min), and 100% B (53–60 min). The separated peptides were analyzed using a Q-Exactive HF-X mass spectrometer in data-dependent acquisition (DDA) mode with the following parameters: 60 min acquisition time, positive ion mode, 350–1800 *m*/*z* scan range, 60,000 resolution for MS1 (AGC target 3e6), and 20 most intense ions selected for MS2 (15,000 resolution, AGC target 1e5, HCD activation, 1.6 m/z isolation window, 28% collision energy). The acquired data were processed using MaxQuant software (version 2.4.14.0) for peptide identification.

#### 2.4.2 In silico screening of bioactive peptides

A systematic bioinformatics approach was employed to evaluate the potential bioactivity and safety of the identified peptide sequences through multidimensional prediction. First, the Peptide Ranker program (http://distilldeep.ucd.ie/PeptideRanker/) was used to predict potential bioactivity based on a machine learning algorithm that integrates multiple peptide features, with scores ranging from 0 to 1 (higher scores indicating greater bioactivity potential) (Ouyang et al., 2024). To assess safety, the Toxin Pred tool (http://crdd.osdd.net/raghava//toxinpred/) was applied to evaluate potential toxicity risks using a support vector machine-based model to identify toxicity-related sequence patterns (Zhang et al., 2024). AllergenFP (https://www.ddg-pharmfac.net/AllergenFP/) was utilized to predict allergenic structural features or sequence fragments, thereby reducing immunogenicity risks and optimizing targeted delivery efficiency (Hu et al., 2025). Key physicochemical properties were analyzed using PepDraw (http://www.tulane.edu/∼biochem/WW/PepDraw/index.html) for structural visualization, combined with PepCalc (http://pepcalc.com/) to compute parameters such as isoelectric point and hydrophobicity (Hu et al., 2024). Sequence alignment with known bioactive peptides was performed using the BIOPEP-UWM database (https://biochemia.uwm.edu.pl/biopep/peptide_data.php), which contains over 4000 experimentally validated functional peptides (Xiao et al., 2024). Finally, the PreAIP algorithm (http://kurata14.bio.kyutech.ac.jp/PreAIP/index.php) was employed to predict and screen peptide sequences with potential anti-inflammatory activity (selection threshold >0.468) (de Azevedo et al., 2024). Cross-validation using these methods ensured the reliability of the prediction results.

### 2.5 Peptide synthesis

The screened anti-inflammatory peptides were synthesized via solid-phase synthesis by GenScript Co., Ltd. (Jiangsu, China). The purified peptide was characterized using analytical techniques such as mass spectrometry and HPLC to confirm its identity and purity (≥98%).

### 2.6 Determination of cell relative proliferation rate

The RAW264.7 macrophages, preserved in our laboratory, was routinely maintained in complete culture medium consisting of 89% DMEM high-glucose medium, 10% fetal bovine serum (FBS), and 1% penicillin-streptomycin solution. Cells were cultured at 37 °C in a humidified atmosphere containing 5% CO₂.

For the cytotoxicity assessment, RAW264.7 macrophages in logarithmic growth phase were seeded in 96-well plates at a density of 6 × 10^5^ cells/mL (100 μL/well). The experimental design included three groups: blank control (100 μL complete medium without cells), negative control (cells without peptide treatment), and treatment groups (cells exposed to synthetic peptides at concentrations ranging from 50 to 400 μg/mL). Peripheral wells were filled with PBS to minimize evaporation. After 24 h of adhesion, cells were treated with peptide solutions for an additional 24 h. Subsequently, 100 μL of culture medium containing 10% CCK-8 reagent was added to each well, followed by 1 h incubation at 37 °C. Absorbance was measured at 450 nm using a SpectraMax190 microplate reader (Molecular Devices, USA) with six replicates (*n* = 6). Cell relative proliferation rates were calculated to evaluate peptide cytotoxicity and determine appropriate concentrations for subsequent experiments.

### 2.7 Determination of nitric oxide production

NO levels were determined by measuring nitrite concentration in cell supernatants using the Griess assay (Zheng et al., 2025). RAW264.7 macrophages (6 × 10^5^ cells/mL, 100 μL/well) were seeded in 96-well plates and cultured for 24 h to allow adhesion. The experiment included three groups: a blank control group, a negative control group (same as in Section 2.6), and an experimental group (containing cells with synthetic peptide solutions at concentrations of 100–400 μg/mL). Following LPS stimulation (1 μg/mL, 100 μL/well, 24 h) in blank control and treatment groups, peptide solutions (100–400 μg/mL) were added to treatment groups while blank control groups received equal volumes of DMEM high-glucose medium. After 24 h incubation, 50 μL aliquots of supernatant from each well were mixed sequentially with 50 μL each of Griess Reagents I and II. Absorbance was measured at 540 nm, with nitrite concentration calculated using a standard curve (Y = 0.0051×-0.0067, R^2^ = 0.9991) to quantify NO production.

### 2.8 Cytokines assay

Cell culture supernatants were collected from RAW264.7 macrophages treated according to the method described in Section 2.7. The concentrations of cytokines (TNF-α, IL-1β, and IL-6) were determined using commercial ELISA kits following the manufacturer's protocols (Zhao et al., 2023), (Zhao et al., 2025). All assays were performed in 96-well microtiter plates with six replicate wells per sample (*n* = 6). Absorbance was measured using a SpectraMax190 microplate reader (Molecular Devices, USA), and cytokine concentrations were calculated based on standard curves.

### 2.9 Molecular docking analysis

The three-dimensional structures of peptide ligands were obtained in SDF format from the PubChem database, while the crystal structures of TLR2 and TLR4 were downloaded from the Protein Data Bank (PDB). Protein structures were preprocessed using PyMOL 2.1.0 to remove water molecules and extraneous small molecule ligands. Subsequent preparation of the target proteins was performed with AutoDock Tools 1.5.6 (Xu, Wang, et al., 2025), including the addition of hydrogen atoms and calculation of partial atomic charges, before conversion to the PDBQT file format for molecular docking simulations.

The molecular docking simulations were performed using the Vina 2.0 program integrated within the Pyrx software platform, with TLR2 and TLR4 proteins as receptors and anti-inflammatory peptides as ligands. The binding affinity (expressed in kcal/mol) was calculated for each complex, where lower energy values indicated more stable interactions. Molecular docking results file were visualized and analyzed using PyMOL 2.1.0, while two-dimensional interaction diagrams were generated with Discovery Studio 2020 Client (https://discover.3ds.com/discovery-studio-visualizer-download) to illustrate key molecular interactions.

### 2.10 Statistical analysis

Data were derived from at least six independent experiments and expressed as mean ± standard deviation (SD). Statistical significance was determined by one-way analysis of variance (ANOVA) followed by Duncan's test using IBM SPSS Statistics 26, with a significance threshold of *P* < 0.05. Graphical representations were generated using Origin 2022 software (OriginLab Corporation, USA).

## 3 Results and discussions

### 3.1 Molecular weight distribution of sea cucumber gonad-derived peptides

**Fig. 1:**
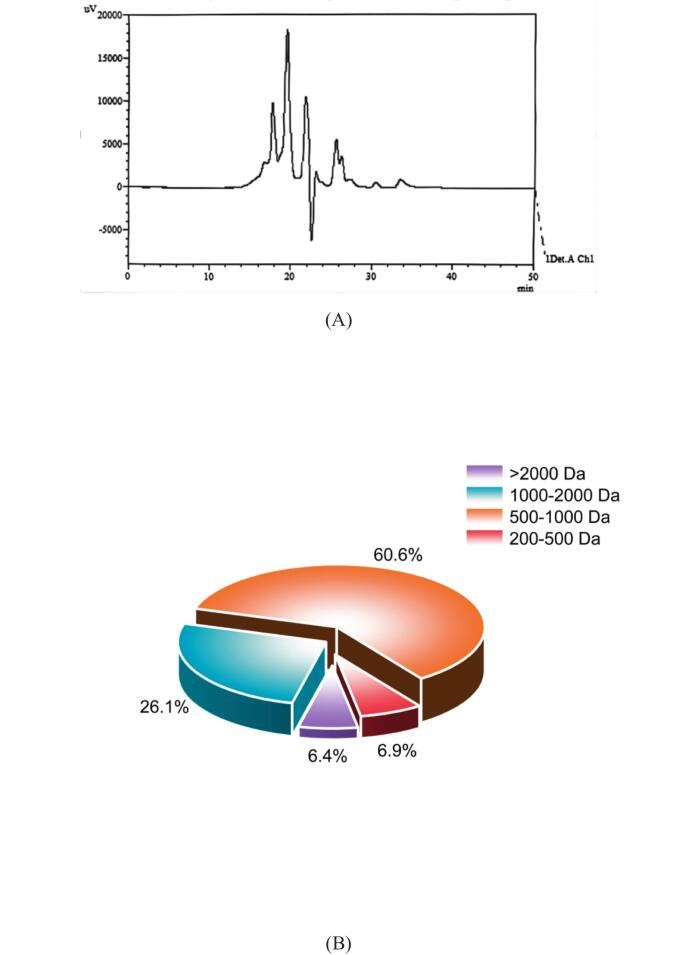
Liquid chromatography profile (A) and molecular weight distribution profile (B) of the 200–3000 Da fraction.

The molecular weight and length of peptides are critical structural factors determining their bioactivity (Sun, Chen, et al., 2025). Studies have demonstrated that low-molecular-weight peptides not only effectively resist degradation by digestive proteases (though this characteristic highly depends on peptide sequence and structure), but also exhibit enhanced their permeability across the intestinal epithelium, thereby demonstrating high bioavailability (Shahosseini et al., 2022). Therefore, the present study focused on peptide fractions below 2 kDa, which accounted for 93.6% of the entire peptides prepared, especially since the molecular weight of almost two - thirds of peptides is <1 kDa **(**Fig. 1**)**. These ratios were significantly higher than the those reported for bovine whey protein hydrolysate (44%) and yak bone collagen hydrolysate (64%) (Li, Liu, et al., 2025; Ma et al., 2016). The high degree of hydrolysis resembles that of highly active marine protein hydrolysates, such as the <1 kDa fractions of oyster peptides (62.7%) and sturgeon head peptides (56.5%) (Islam et al., 2023; Jiang et al., 2024b). Previous studies have confirmed that low-molecular-weight peptides (< 1 kDa) exhibit superior bioactivity. For instance, the <1 kDa fraction of mung bean protein hydrolysate demonstrated significantly stronger inhibition of LPS-induced inflammatory cytokines compared to larger molecular-weight fractions (Diao et al., 2022), consistent with the previous findings that low-molecular-weight peptides possessed greater antioxidant, anti-inflammatory, and immunomodulatory activities (Fang et al., 2021; Jeong et al., 2024; Martínez-Sánchez et al., 2019). The high proportion (67.5%) of the <1 kDa fraction of sea cucumber gonad peptides not only suggests potentially superior intestinal absorption properties compared to other protein hydrolysates, but also provides an important structural basis for their potential anti-inflammatory and physiological regulatory functions.

### 3.2 Physicochemical properties of sea cucumber gonad peptides

**Fig. 2:**
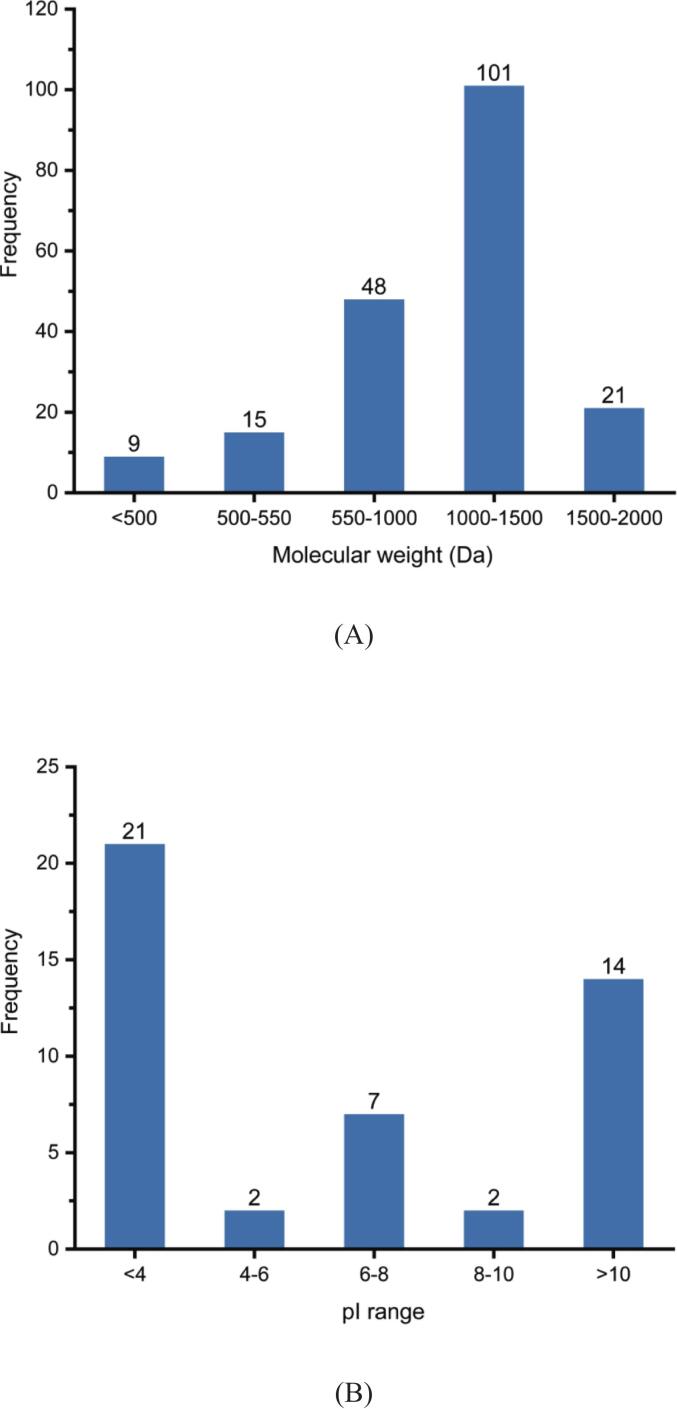
Physicochemical properties of peptides: molecular weight (A), pI (B).

High-throughput sequencing of the sea cucumber gonad peptide (SCGP) fraction via LC-MS/MS identified 199 peptide sequences, including critical parameters such as amino acid sequences, parent proteins, peptide spectrum matches (PSMs), and molecular weights **(Table S1)**. The peptide length ranged from 5 to 27 amino acid residues, with molecular weights spanning 439–2414 Da, out of which 74.9% of the peptides fell within the 550–1500 Da range **(**Fig. 2A**)**, further confirming the predominance of low-molecular-weight peptides in the fraction.

To assess their potential bioactivity, PeptideRanker was employed for activity prediction, revealing that 46 peptides scored significantly above the 0.5 activity threshold (Garzón et al., 2022), indicating a high probability of biological activity. These bioactive peptides exhibited diverse isoelectric point (pI) distributions: 21 were strongly acidic (pI <4), 9 were weakly acidic/neutral (4 < pI <8), and 16 were basic (pI >8), with net charges consistently ranging between −1 and + 1 **(**Fig. 2B**)**.

Of particular interest, hydrophobic interactions play a crucial role in TLR2/4 receptor activation by enhancing ligand-receptor binding affinity, thereby promoting anti-inflammatory signaling pathways (Klink et al., 2012). To evaluate this mechanism, the hydrophobic characteristics of the 46 bioactive peptides were systematically analyzed. Results indicated that all bioactive peptides exhibited hydrophobicity, containing hydrophobic amino acids such as glycine (Gly), alanine (Ala), valine (Val), leucine (Leu), isoleucine (Ile), proline (Pro), phenylalanine (Phe), tryptophan (Trp), and methionine (Met). The proportion of hydrophobic residues ranged from 14.29% to 100%, with 40% of peptides exceeding 50% hydrophobicity **(**Table 1**)**. Previous studies have demonstrated that highly hydrophobic peptides (e.g., AMKPWIQPK) can bind immune receptors (e.g., TLR2/4) via hydrophobic interactions and modulate inflammatory responses (Jiang et al., 2025). Based on these results, the hydrophobic peptides identified in this study are speculated to possess structural characteristics that may confer potential anti-inflammatory activity (Jiang et al., 2024a).

**Table 1:** Proportions of hydrophobic, acidic, and basic amino acid residues in peptides.

Peptides	Hydrophobicity %	Acidic %	Basic %
GPRGF	40	0	20
MPPPP	100	0	0
PGHPF	60	0	20
GPMGP	60	0	0
FPGQL	60	0	0
LPGPP	80	0	0
GDRGF	20	20	20
VPFPR	80	0	20
GPLGP	60	0	0
NPWGQ	40	0	0
GPFGQ	40	0	0
LGGPL	60	0	0
APMNP	80	0	0
SEGPF	40	20	0
LPFQR	60	0	20
APLLL	100	0	0
GPAGPQGP	50	0	0
GQRGPAGPTGPTGPAGATGERGPSGPQ	33.33	3.7	7.41
FDGPEGPRGPPGSEGRQG	27.78	16.67	11.11
GQRGPAGPTGPTGP	35.71	0	7.14
GPAGPTGPTGPA	50	0	0
ATGPQGQQGSRGERGPEGQQG	14.29	9.52	9.52
GGGTGSGMGTL	18.18	0	0
TQQMF	40	0	0
VPLGM	80	0	0
GPVGL	60	0	0
APLNP	80	0	0
GPDASEGDRSRGGGPGR	17.65	17.65	17.65
GQRGPAGPTGP	36.36	0	9.09
ATGPQGPAGQRGPAGPTGPTGPA	43.48	0	4.35
ATGPQGQQGSRGERGPEGQQGQAG	16.67	8.33	8.33
GQRGPAGPTGPT	33.33	0	8.33
GQRGPAGPTGPTGPAG	37.5	0	6.25
AGPTGPTGP	44.44	0	0
DMKVF	60	20	20
ATGPQGPAGQRGPAGPTGPTGPAG	41.67	0	4.17
AIADLNDPKGSSGTAIKK	38.89	11.11	16.67
QRGPAGPTGPTGPA	42.86	0	7.14
GSTGPAGPQGPAGDR	33.33	6.67	6.67
GNQGPRGGPGETGK	14.29	7.14	14.29
GQRGPAGPTGPTGPAGA	41.18	0	5.88
TGVDNPGHPF	40	10	10
GPDGQAGERGPRGPQ	26.67	13.33	13.33
LPLKL	80	0	20
AGPTGPTGPAG	45.45	0	0
PSNLGTGLR	33.33	0	11.11

### 3.3 Screening of sea cucumber gonad peptides with potential anti-inflammatory activity

**Fig. 3:**
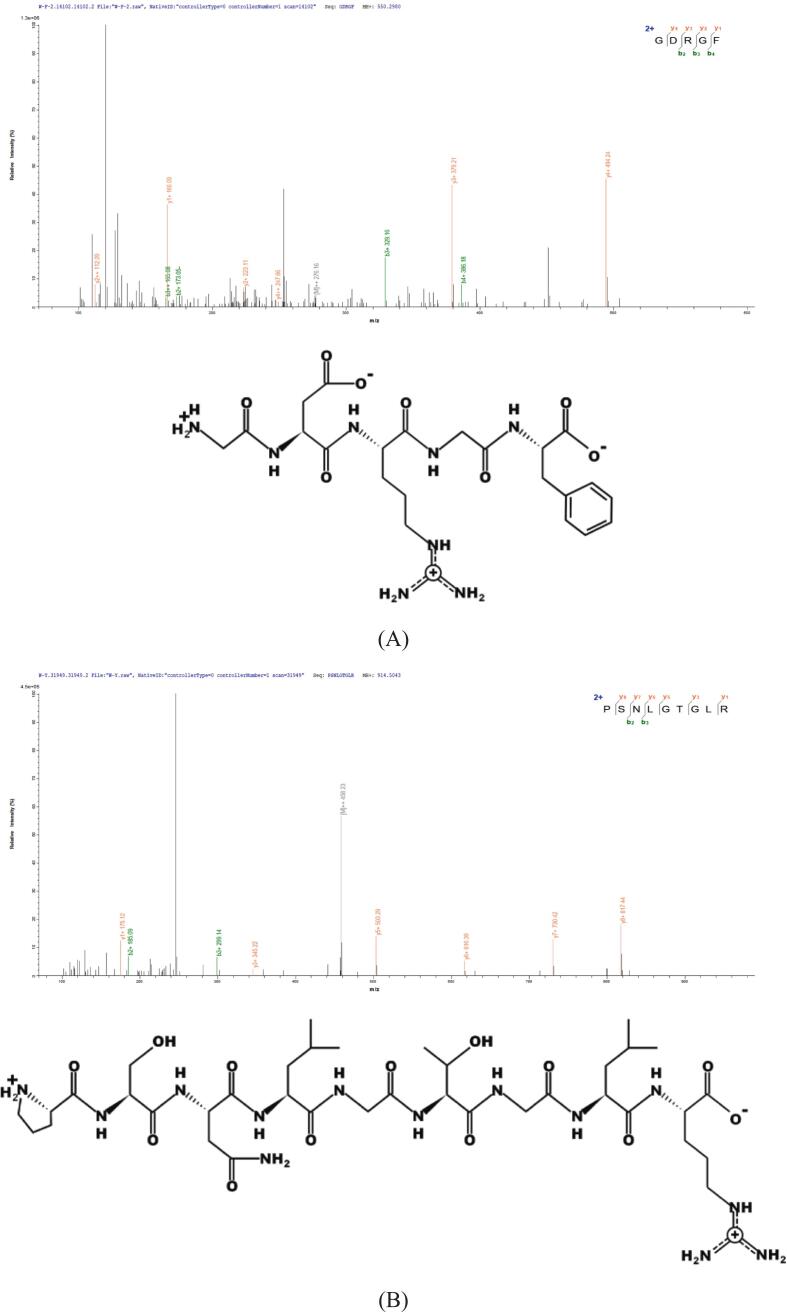

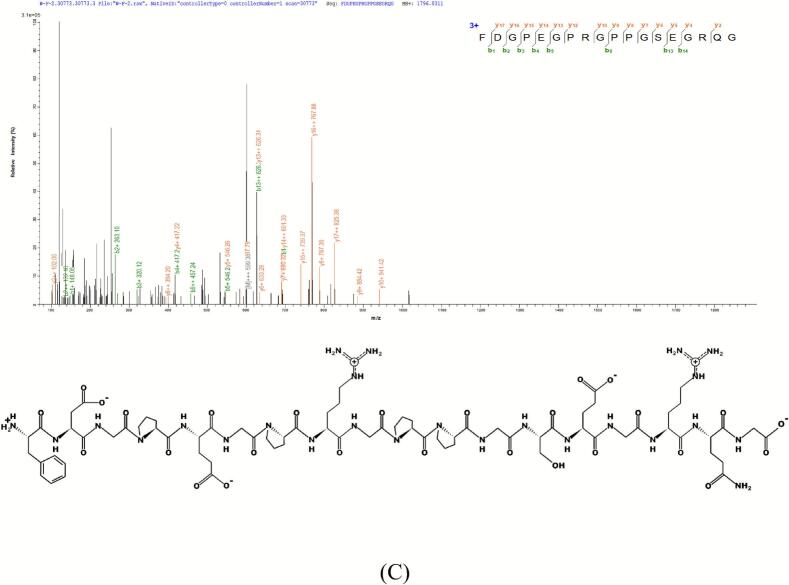
The MS/MS spectra and structures of the identified peptide: (A) GDRGF, (B) PSNLGTGLR, and (C) FDGPEGPRGPPGSEGRQG.

Toxicity, hydrophobicity, and allergenicity were evaluated to screen peptides with promising bioactivity and less safety concern. Toxicity assessment represents a critical step in virtual screening to ensure the safety of candidate peptides for bioactive applications (Yu et al., 2021). In this study, ToxinPred analysis of the 46 candidate peptides confirmed that none exhibited toxicity. Subsequently, PreAIP was employed to predict anti-inflammatory activity (Zhu et al., 2022), identifying three peptides with scores >0.468 (Jiang, Li, et al., 2024b)， GDRGF, PSNLGTGLR, and FDGPEGPRGPPGSEGRQG (Table 2). These peptides contained hydrophobic residues at proportions of 60.00%, 61.10%, and 55.60%, respectively. Hydrophobic interactions play a crucial role in TLR4-MD2 dimerization (Lopez et al., 2024b). Increased hydrophobicity enhances peptide binding affinity to TLR4/MD2, thereby potentiating anti-inflammatory effects. Similar results were also seen for anti-inflammatory peptides derived from crocodile sources (AKLDLEEVIK and DFLDLPSIER), which contained 50% hydrophobic amino acid residues (Hu et al., 2025). Furthermore, in silico analysis confirmed that the three anti-inflammatory peptides were non-allergenic, with molecular masses ranging from 550.56 to 1796.85 Da **(**Table 1**)**. Notably, these peptides have not been previously reported, indicating structural novelty. Their mass spectra and peptide structures are illustrated in Fig. 3. These findings highlight the potential of these novel peptides as safe and effective anti-inflammatory agents.

**Table 2:** Predicted ranking of the anti-inflammatory peptides.

Peptides	PeptideRanker	Toxicity	Mass(g/mol)	PI	Hydrophobicity %	Allergen	PreAIP
GPRGF	0.962121	Non-toxic	550.56	6.64	20.00	Non-alllergen	0.500
PSNLGTGLR	0.591755	Non-toxic	914.02	11.29	33.33	Non-alllergen	0.514
FDGPEGPRGPPGSEGRQG	0.684086	Non-toxic	1796.85	4.32	27.78	Non-alllergen	0.585

### 3.4 Proliferative effect of anti-inflammatory peptides on RAW264.7 macrophages

**Fig. 4:**
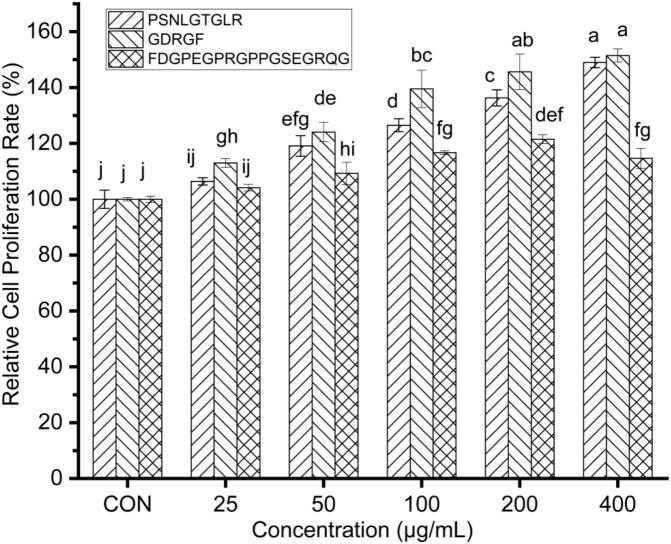
Effects of the anti-inflammatory peptides on the relative proliferation rate of RAW264.7 macrophages. Different lowercase letters in the figure indicate statistically significant differences (*P* < 0.05).

The CCK-8 assay, a well-established method for determining cytotoxicity (Wang et al., 2025), was employed to evaluate the effects of three synthetic peptides (PSNLGTGLR, GDRGF, and FDGPEGPRGPPGSEGRQG) on the relative proliferation rate of RAW264.7 macrophages. As shown in Fig. 4, all peptides significantly enhanced cell proliferation (*P* < 0.05) in a concentration-dependent manner within the 25–400 μg/mL range. Among them, PSNLGTGLR and GDRGF exhibited the strongest proliferative effects at 400 μg/mL, increasing relative proliferation rates to (149.0 ± 1.86)% and (151.5 ± 2.33)%, respectively. FDGPEGPRGPPGSEGRQG reached its peak effect at 200 μg/mL, achieving a relative proliferation rate of (121.5 ± 1.57)%. Notably, these peptides demonstrated superior proliferative activity compared to the egg white-derived peptide PA11, which only showed a 105.6% proliferation rate at 1000 μg/mL (Wanthong et al., 2024).

Importantly, none of the peptides exhibited cytotoxicity at the tested concentrations (25–400 μg/mL). Based on these results, a concentration range of 100–400 μg/mL was selected for subsequent experiments. These findings highlight the potential of these synthetic peptides as safe and effective macrophage proliferative agents, warranting further investigation into their underlying mechanisms.

### 3.5 Validation of anti-inflammatory activity of identified peptides

#### 3.5.1 Inhibitory effects of anti-inflammatory peptides on NO secretion in RAW264.7 macrophages

**Fig. 5:**
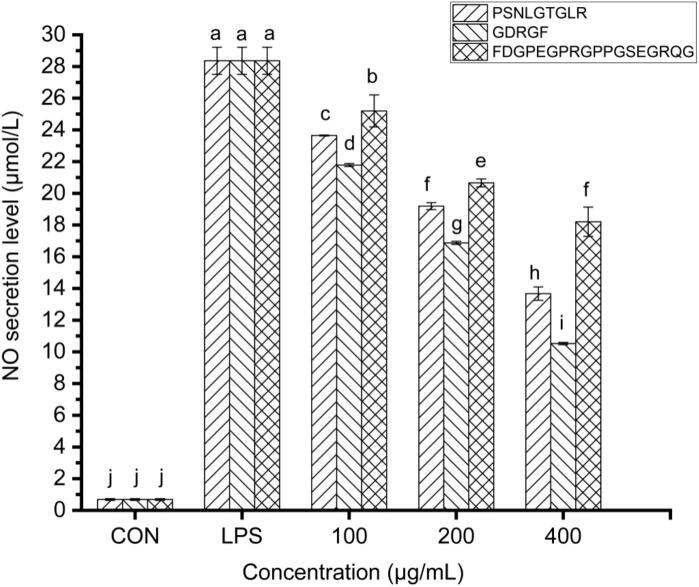
Effects of the anti-inflammatory peptides on NO secretion in RAW 264.7 cells. Different lowercase letters in the figure indicate statistically significant differences (*P* < 0.05).

The NO induced by inducible nitric oxide synthase (iNOS), as a key regulator of inflammatory responses, is involved in the modulation of cell growth and development (Song et al., 2019), while its excessive release can lead to toxicity and pro-inflammatory effects, thereby causing tissue damage (Palmieri et al., 2020). Therefore, NO levels can directly reflect the inflammatory state of cells (Yang et al., 2024). In the LPS-stimulated RAW264.7 macrophage model, NO release was significantly increased, reaching 28.4 ± 0.86 μmol/L (*P* < 0.05) **(**Fig. 5**)**. However, sea cucumber gonad-derived peptides PSNLGTGLR, GDRGF, and FDGPEGPRGPPGSEGRQG significantly inhibited NO release, with inhibition rates of 16.62%–51.80%, 23.20%–62.89%, and 11.15%–35.81%, respectively (*P* < 0.05) **(**Fig. 5**)**. Compared with previously reported anti-inflammatory peptides, GDRGF exhibited more pronounced NO inhibitory activity. Its inhibitory effect was superior to that of marine-derived perch anti-inflammatory peptides DAPAPPSQLEHIRAA and AADGPMKGILGY (20.00%) (Chen et al., 2021), as well as crocodile-derived anti-inflammatory peptides (Hu et al., 2025). Additionally, it outperformed animal-derived peptides such as porcine liver-derived peptides (25.10% inhibition at 5 mg/mL) (Miao et al., 2025) and porcine bone-derived peptides (20.62% inhibition at 1 mg/mL) (Hao et al., 2023), demonstrating significantly enhanced efficacy. Furthermore, the efficacy of these sea cucumber gonad peptides at relatively low concentrations (e.g., GDRGF achieving 62.89% inhibition at 400 μg/mL) compares favorably with or exceeds that of many reported peptides from other marine sources, such as peptides derived from clams and oysters (Ji et al., 2024; Lin et al., 2024), which often require higher concentrations to achieve comparable effects. These results confirm the anti-inflammatory potential of sea cucumber gonad-derived peptides, providing important experimental evidence and candidate molecules for the development of novel, highly effective anti-inflammatory peptide drugs. Compared to known anti-inflammatory peptides, the advantage of GDRGF lies not only in its higher inhibition rate but also potentially in its structural characteristics. Anti-inflammatory peptides from different sources exhibit variations in amino acid composition, sequence length, and spatial structure, which may influence their binding affinity and functional efficiency with target molecules (Kim et al., 2005).

#### 3.5.2 Anti-inflammatory peptides inhibit pro-inflammatory cytokine secretion in RAW264.7 macrophages

**Fig. 6:**
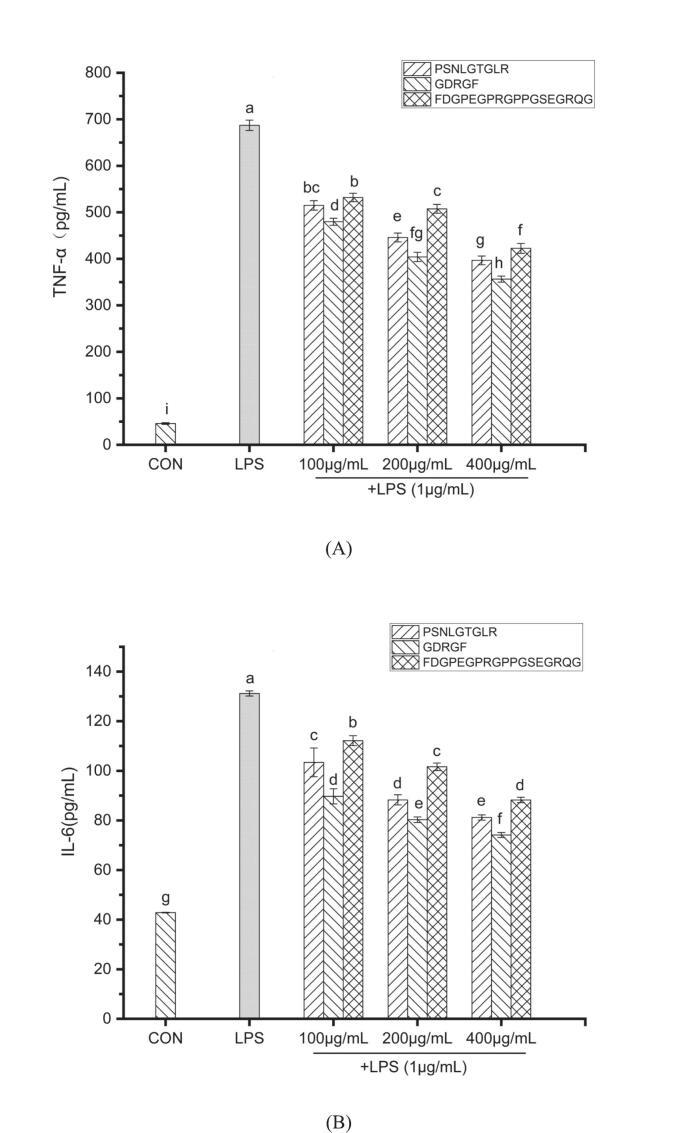

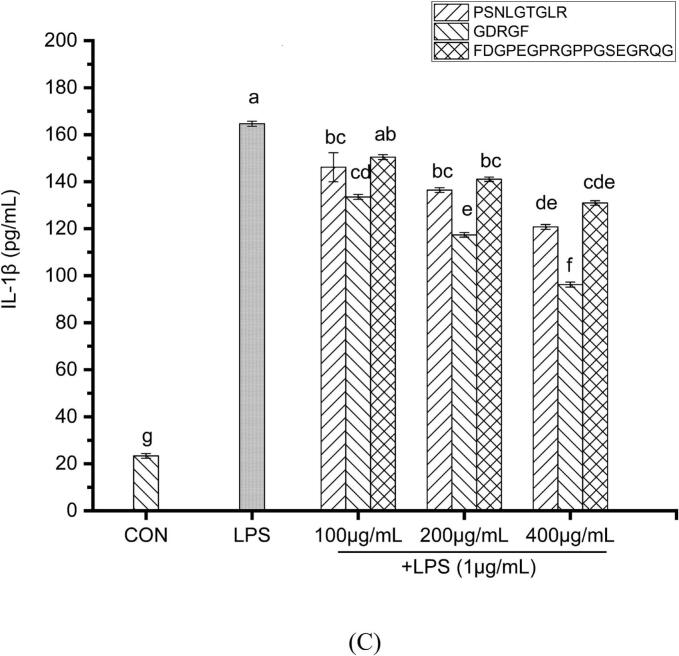
The effect of the anti-inflammatory peptides on the secretion of pro-inflammatory cytokines in LPS-stimulated RAW264.7 cells. (A) TNF-α, (B) IL-6, and (C) IL-1β. Different lowercase letters in the figure indicate statistically significant differences (*P* < 0.05).

Macrophage activation is a critical component of the body's immune defense. Upon recognition of endotoxins such as LPS, macrophages trigger an inflammatory signaling cascade, releasing various pro-inflammatory cytokines, including IL-1β, IL-6, and TNF-α, along with other inflammatory mediators (Gao et al., 2020). Excessive macrophage activation and aberrant overexpression of these cytokines induced by LPS stimulation are closely associated with numerous inflammatory diseases (Fu et al., 2021). This study evaluated the inhibitory effects of three sea cucumber gonad-derived peptides (PSNLGTGLR, GDRGF, and FDGPEGPRGPPGSEGRQG) on the production of pro-inflammatory cytokines in LPS-stimulated macrophages at concentrations ranging from 100 to 400 μg/mL **(**Fig. 6**)**.

In the LPS-stimulated control group, the secretion levels of TNF-α, IL-6, and IL-1β were 687.0 ± 11.10 pg/mL, 131.2 ± 1.05 pg/mL, and 164.7 ± 1.07 pg/mL, respectively. Treatment with the three peptides significantly suppressed the release of these cytokines. For PSNLGTGLR, GDRGF, and FDGPEGPRGPPGSEGRQG, TNF-α levels were inhibited by 25.06%–42.28%, 30.16%–48.14%, and 22.58%–38.51% (*P* < 0.05), respectively; the IL-1β inhibition rates were 11.24%–26.65%, 18.92%–41.57%, and 8.61%–20.50% (*P* < 0.05), while the IL-6 inhibition rates were 21.18%–38.11%, 31.62%–43.50%, and 14.51%–32.74% (*P* < 0.05) **(**Fig. 6**)**. Notably, peptide GDRGF exhibited a 43.50% inhibition rate at 400 μg/mL against IL-6, reducing IL-6 levels to 74.12 pg/mL. This inhibitory effect was superior to that of anti-inflammatory peptides derived from bovine hemoglobin (Xin et al., 2024). At the same concentration, the secretion levels of IL-1β were approximately 120.78 pg/mL (PSNLGTGLR), 96.21 pg/mL (GDRGF), and 130.91 pg/mL (FDGPEGPRGPPGSEGRQG) **(**Fig. 6C**)**, consistent with studies demonstrating anti-inflammatory effects through the suppression of pro-inflammatory cytokine production (Kuo et al., 2024).

TNF-α is a key early inflammatory mediator and triggers the inflammatory cascade by activating neutrophils and increasing vascular permeability (Kim et al., 2018). This study revealed that all three peptides significantly inhibited its release, demonstrating their remarkable bioactivity in regulating inflammatory responses. Furthermore, since IL-1β not only directly initiates inflammatory cascades (Nazakat et al., 2025) but also amplifies inflammation by inducing the production of TNF-α and IL-6 (Khunoana et al., 2025), suppressing IL-1β is particularly crucial for controlling the overall inflammatory response.

To summarize, the sea cucumber gonad-derived peptides PSNLGTGLR, GDRGF, and FDGPEGPRGPPGSEGRQG exhibit anti-inflammatory activity in LPS-stimulated macrophages by significantly inhibiting the production of key pro-inflammatory cytokines, including TNF-α, IL-6, and IL-1β.

### 3.6 Interaction of anti-inflammatory peptides with TLR2/TLR4

To further evaluate the binding strength of the three screened high-activity anti-inflammatory peptides (GDRGF, PSNLGTGLR, FDGPEGPRGPPGSEGRQG) to TLR2/4, we conducted a comparative analysis with literature data on known TLR ligands (e.g., LPS). Molecular docking results demonstrated that the binding energies of the three anti-inflammatory peptides to both TLR2 and TLR4 (all ≤ −7.0 kcal/mol) were significantly superior to that of LPS with TLR4 (−5.7 kcal/mol) (Budiarti et al., 2024; Sun, Hui, et al., 2025). In TLR2-related studies, the docking scores of these peptides were also markedly lower than the docking score of PSPP-1 with the receptor protein TLR2 (−5.6 kcal/mol) (Ji et al., 2021), indicating that these anti-inflammatory peptides exhibit stronger binding affinity to Toll-like receptors (Zhao et al., 2021). These findings robustly confirm the high-affinity binding between the three peptides and TLR2/4 receptors.

**Fig. 7:**
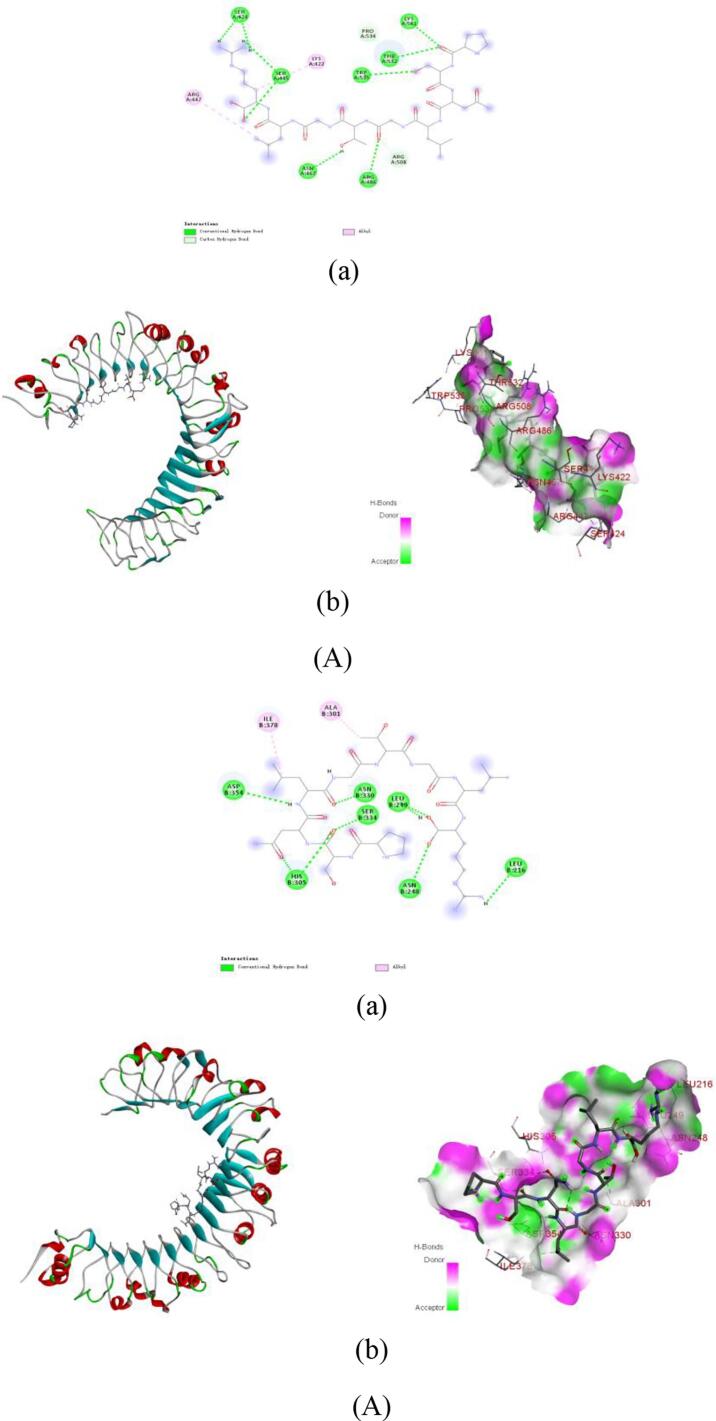

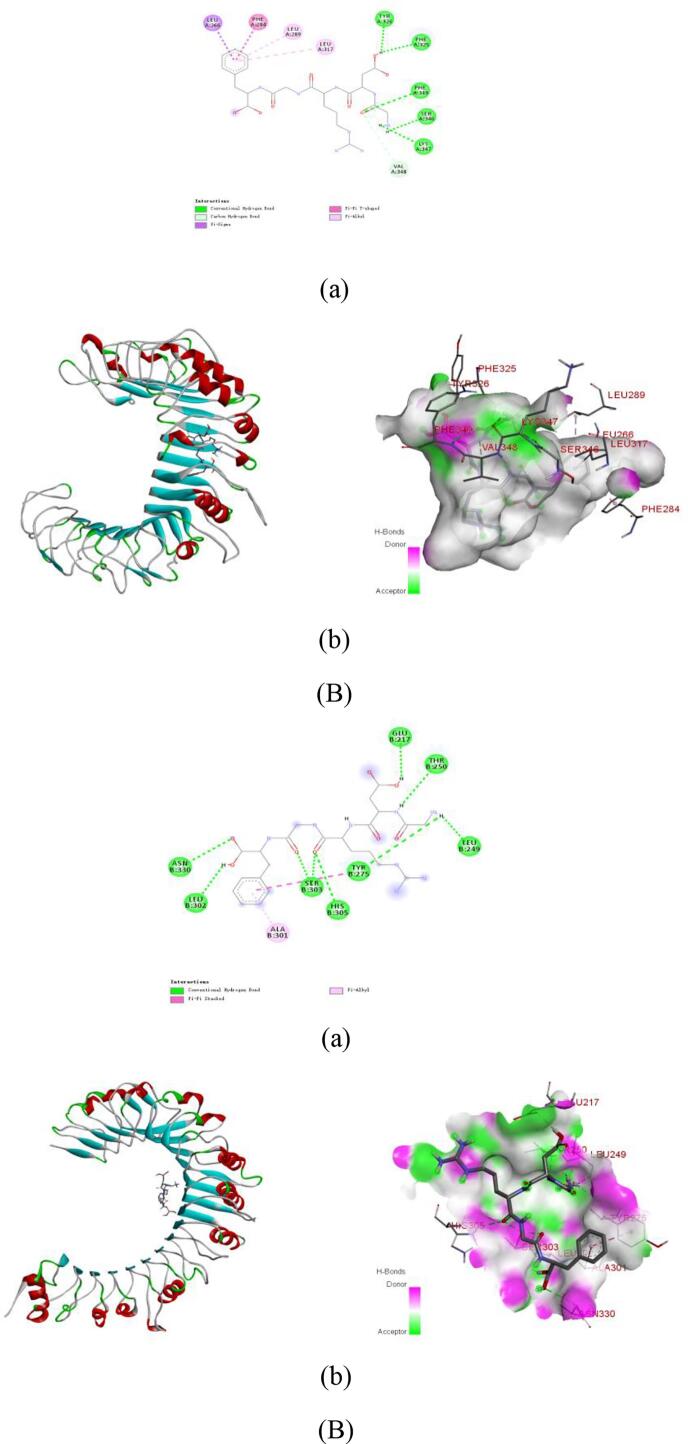

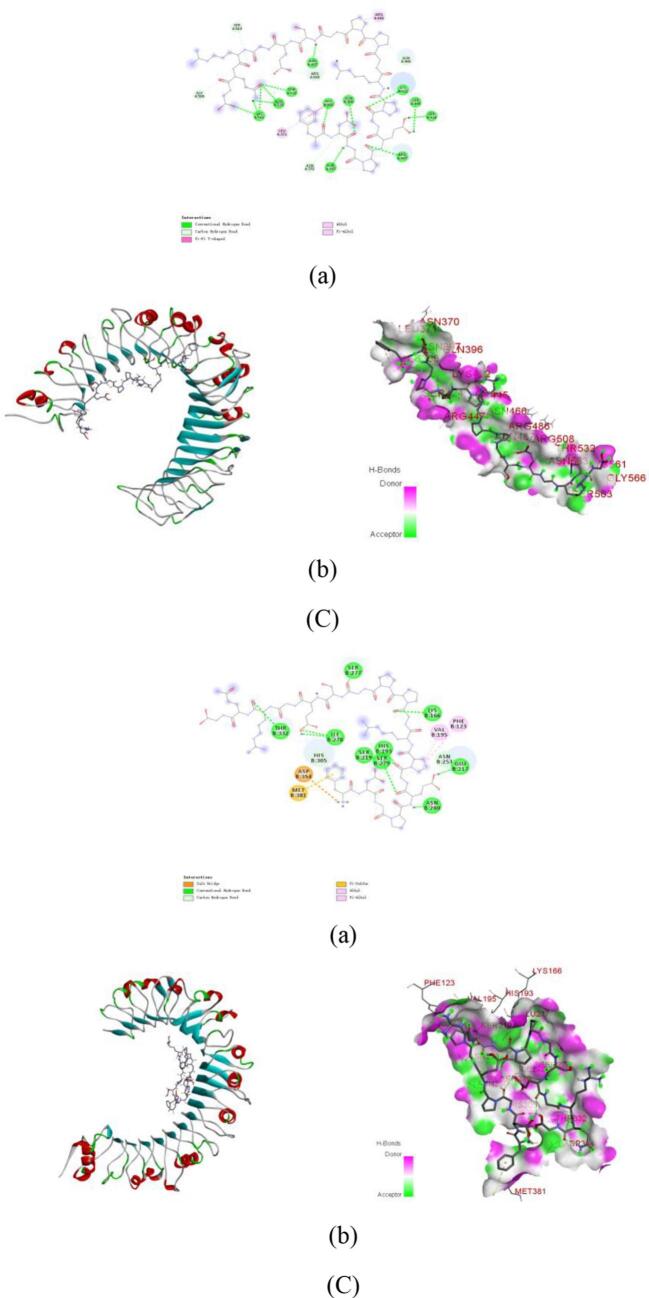
Molecular docking of the anti-inflammatory peptides with TLR4. (A) PSNLGTGLR-TLR4. a: 2D diagram of peptide-target protein interactions; b: 3D diagram of peptide-target protein binding. Molecular docking of the anti-inflammatory peptides with TLR4. (B) GDRGF-TLR4. a: 2D diagram of peptide-target protein interactions; b: 3D diagram of peptide-target protein binding. Molecular docking of the anti-inflammatory peptides with TLR4. (C) FDGPEGPRGPPGSEGRQG-TLR4. a: 2D diagram of peptide-target protein interactions; b: 3D diagram of peptide-target protein binding.

PyMol visualization and interaction analysis (Fig. 7**,** Table 3) elucidated that the stability of peptide-receptor complexes relies on extensive hydrogen bond networks and diversified hydrophobic interactions, including alkyl, π-alkyl, π-σ, and π-π stacking. In the TLR2 system (Table 2), GDRGF formed six hydrogen bonds with Tyr326, Phe325, Phe349, Ser346, Lys347, and Val348, along with hydrophobic interactions involving Leu289, Leu317, Leu266, and Phe284. PSNLGTGLR established nine hydrogen bonds with Ser424, Ser445, Asn467, Arg486, Trp535, Thr532, Lys561, Pro534, and Arg508, supplemented by alkyl interactions with Arg447 and Lys422. Notably, FDGPEGPRGPPGSEGRQG exhibited the strongest binding, forming 16 hydrogen bonds with Gly566, Ser563, Lys561, Asn533, Thr532, Asn487, Arg508, His398, Gln396, Asn466, Lys422, Ser445, Ser424, Asn370, Asn397, and Arg447, along with alkyl/π-alkyl interactions with Leu371 and Arg486.

**Table 3:** Molecular docking energy and binding sites of the anti-inflammatory peptides with TLR2 and TLR4.

Peptides	Receptors	Binding Affinity(kcal/mol)	Binding sites of hydrogen bonds	Binding sites of hydrophobic interactions	Salt bridge
GDRGF	TLR2	−7.3	Tyr326, Phe325, Phe349, Ser346, Lys347, Val348	Leu289, Leu317, Phe284	
	TLR4	−7.6	Asn330, Leu302, Ser303, His305, Tyr275, Leu249, Thr250, Glu217	Ala301	
PSNLGTGLR	TLR2	−8.0	Ser424, Ser445, Asn467, Arg486, Trp535, Thr532, Lys561, Pro534, Arg508	Arg447, Lys422	
	TLR4	−7.0	Asp354, His305, Asn330, Ser334, Leu249, Asn248, Leu216	Ile378, Ala301	
FDGPEGPRGPPGSEGRQG	TLR2	−7.5	Gly566, Ser563, Lys561, Asn533, Thr532, Asn487, Arg508, His398, Gln396, Asn466, Lys422, Ser445, Ser424, Asn370, Asn397, Arg447	Leu371, Arg486	
	TLR4	−7.3	Thr332, Ser277, Ile278, His305, Ser219, His193, Ser279, Asn280, Lys166, Asn253, Glu217	Val195, Phe123	Asp354

The stability of peptide-receptor complexes in the TLR4 system relies on the synergistic effects of hydrogen bond networks and diversified hydrophobic interactions, including alkyl, π-alkyl, π-σ, and π-π interactions. Specifically, GDRGF forms eight hydrogen bonds with Asn330, Leu302, Ser303, His305, Tyr275, Leu249, Thr250, and Glu217, along with a π-alkyl hydrophobic interaction with Ala301. PSNLGTGLR establishes seven hydrogen bonds with Asp354, His305, Asn330, Ser334, Leu249, Asn248, and Leu216, complemented by alkyl interactions with Ile378 and Ala301. Meanwhile, FDGPEGPRGPPGSEGRQG exhibits a unique binding pattern involving eleven hydrogen bonds with Thr332, Ser277, Ile278, His305, Ser219, His193, Ser279, Asn280, Lys166, Asn253, and Glu217, alongside alkyl and π-alkyl interactions with Val195 and Phe123, a salt bridge with Asp354, and a π‑sulfur interaction with Met381. These findings align with previously reported TLR-receptor interaction characteristics (Xu et al., 2024; Xu, Yi, et al., 2025), providing critical structural insights for the rational design of anti-inflammatory peptides.

## 4 Discussion

The ‘in silico-guided bioactive peptide screening’ strategy employed in this study demonstrated remarkable efficiency compared to the conventional ‘random enzymatic hydrolysis–separation–activity screening’ approach. Traditional methods involve lengthy procedures, extensive blind screening, and complex chromatographic separations, which are time-consuming, labor-intensive, and lack clear objectives. In contrast, our strategy first predicts potential bioactive peptides through bioinformatics tools, followed by targeted screening via molecular docking, significantly narrowing the candidate pool. This approach avoids extensive ineffective experimental efforts, rapidly focuses on the most promising peptides, and substantially reduces both the time and cost of screening, thereby demonstrating high efficiency in bioactive peptide discovery. The successful application of this strategy to rapidly identify three candidate anti-inflammatory peptides from complex sea cucumber gonad hydrolysates exemplifies its efficiency.

This study identified three novel anti-inflammatory peptides from sea cucumber gonads. Compared with previously reported marine-derived anti-inflammatory peptides, such as oyster peptides and sturgeon head peptides, the identified peptides exhibit multiple distinctive advantages, further supporting the potential of sea cucumber gonads as a source of marine bioactive peptides. In terms of raw material utilization, sea cucumber gonads represent an underutilized by-product of aquatic processing. In contrast to oyster peptides, which rely on primary edible parts, the use of this by-product offers notable advantages in sustainability and industrial production costs, thereby opening a new avenue for the high-value utilization of marine biological resources.

The molecular weight distribution profile of sea cucumber gonad peptides provides a structural basis for their high bioavailability (Que et al., 2024). Over 93% of the components have molecular weights below 2 kDa, with nearly 70% consisting of ultra-small peptides under 1 kDa. This proportion is significantly higher than that observed in other marine comparative samples. Consequently, these peptides are likely to exhibit enhanced resistance to digestive enzymes and efficient transit across the intestinal barrier(Aquino et al., 2024), conferring an inherent advantage for their direct anti-inflammatory efficacy in vivo. The superior bioactivity of sea cucumber gonad peptides was further confirmed by in vitro assessments. Among the three identified anti-inflammatory peptides, GDRGF exhibited significantly stronger anti-inflammatory effects than previously reported marine-derived anti-inflammatory peptides, including those from perch and crocodile sources, demonstrating outstanding advantages in both cellular safety and active concentration adaptability.

Molecular docking results provide mechanistic insights into the uniqueness of sea cucumber gonad peptides. Their binding energies with TLR2/4 receptors were all ≤ −7.0 kcal/mol, with up to 16 hydrogen bonds formed, along with specific interactions such as salt bridges and π‑sulfur bonds. The stronger receptor-binding affinity and more complex interaction patterns may be key reasons for the superior anti-inflammatory activity of these peptides, which are also closely associated with their distinctive arginine distribution and hydrophobic characteristics. All three anti-inflammatory peptides are enriched with key functional amino acid residues arginine (R) and lysine (K) (Lee et al., 2024). Among them, GDRGF and FDGPEGPRGPPGSEGRQG contain arginine residues within their peptide chains, while PSNLGTGLR features an arginine residue at the C-terminus. This distribution pattern aligns with studies demonstrating that the presence of basic amino acids (e.g., R, K) at the N-terminus, C-terminus, or specific internal positions can significantly enhance anti-inflammatory activity (Guha & Majumder, 2019); (Mudgil et al., 2019). Mechanistically, arginine/lysine residues exert anti-inflammatory effects through dual pathways: (1) suppressing the production of the key pro-inflammatory mediator nitric oxide (NO) (Jegani et al., 2025), and (2) in the case of C-terminal arginine, specifically binding lipopolysaccharide (LPS) to block its induction of inflammatory signaling cascades (Rocca et al., 2021). These findings further support the critical role of arginine and lysine in the functional design of anti-inflammatory peptides.

Although our molecular docking simulations strongly predict the binding potential of the three peptides to TLR2/4 receptors, this remains preliminary in silico evidence. To confirm their anti-inflammatory mechanisms, future studies should experimentally validate their effects on downstream signaling pathways, such as NF-κB and MAPK activation(Guo et al., 2016). For example, Western blot analysis could be employed to detect NF-κB p65 nuclear translocation and IκBα degradation, or phosphorylation-specific antibodies could be used to assess the activation levels of MAPK family members (e.g., p38, JNK, ERK). Additionally, gene assays could directly measure changes in NF-κB transcriptional activity, thereby comprehensively elucidating their anti-inflammatory mechanisms. The docking results from this study provide a clear direction and a robust hypothesis for such in-depth mechanistic investigations.

For the successful application of bioactive peptides in functional foods, their gastrointestinal stability and oral bioavailability are key considerations (Wang et al., 2024b). Based on the analysis of the three identified anti-inflammatory peptides, GDRGF and PSNLGTGLR have relatively low molecular weights and lack consecutive arginine or lysine residues, which may confer better enzymatic stability compared to the larger peptide FDGPEGPRGPPGSEGRQG. However, FDGPEGPRGPPGSEGRQG contains multiple proline and glycine residues, which may impart a certain degree of structural rigidity, potentially enhancing its resistance to proteolytic hydrolysis(Anshulata, & Sarma, B. K., 2025; Graul et al., 2023). All three peptides contain potential trypsin and chymotrypsin cleavage sites, suggesting that they may face degradation challenges upon oral administration(Goettig et al., 2019). Future studies should evaluate the stability of these peptides through simulated gastrointestinal digestion experiments. Regarding absorption and utilization, short peptides may be partially absorbed via peptide transporters in intestinal epithelial cells, while the absorption of longer peptides is more challenging(Shen & Matsui, 2017). Subsequent research will employ Caco-2 cell models to assess their transmembrane permeability, or utilize strategies such as nano-delivery systems to improve their bioavailability.

## 5 Conclusion

This study identified three novel anti-inflammatory peptides (GDRGF, FDGPEGPRGPPGSEGRQG, and PSNLGTGLR) from sea cucumber gonads, with molecular weights ranging from 550 to 1800 Da and unique structural characteristics. In vitro cell experiments confirmed that these peptides were non-toxic to RAW264.7 macrophages and significantly promoted their proliferation at concentrations up to 400 μg/mL. Moreover, they could efficiently inhibit the secretion of NO and pro-inflammatory cytokines in macrophages upon LPC stimulation. Among them, GDRGF exhibited the highest inhibition of NO (62.89%) and IL-6 (43.50%), respectively. Molecular docking analysis revealed that all three peptides bind to TLR2/4 receptors with high affinity (binding energy ≤ −7.0 kcal/mol), primarily through hydrogen bond networks and hydrophobic interactions. The presence of arginine residues either within the peptide chains (GDRGF and FDGPEGPRGPPGSEGRQG) or at the C-terminus (PSNLGTGLR) likely mediates their core anti-inflammatory mechanisms by inhibiting NO production and blocking LPS-triggered signaling pathways. The distinctive molecular composition and potent, multi-target anti-inflammatory efficacy of these sea cucumber gonad peptides underscore the unique value of this marine byproduct as a source of novel bioactive peptides. This study provides a crucial theoretical foundation for value-added utilization of sea cucumber byproducts and the development of marine-derived anti-inflammatory peptides. Further studies employing western blotting and related techniques are warranted to elucidate their regulatory mechanisms in inflammatory signaling pathways.

## CRediT authorship contribution statement

**Zhiqin Zhang:** Writing – original draft, Methodology, Investigation, Data curation, Conceptualization. **Yongke Deng:** Supervision, Software, Methodology, Investigation. **Jingxuan Wang:** Methodology, Data curation. **Peipei Dou:** Writing – review & editing. **Hongbing Fan:** Writing – review & editing, Methodology, Conceptualization. **Xiangquan Zeng:** Formal analysis. **Xinguang Fan:** Supervision. **Haimei Liu:** Writing – review & editing, Supervision, Project administration, Funding acquisition. **Qin Zhao:** Supervision, Funding acquisition.

## Declaration of competing interest

The authors declare that they have no known competing financial interests or personal relationships that could have appeared to influence the work reported in this paper.

## Data Availability

Data will be made available on request.

## Appendix A Supplementary data

Table S1. Peptides identified in the sea cucumber gonad peptide fraction. Image 1
