# Supplementary material for: Screening and identification of novel anti-inflammatory peptides from sea cucumber gonads: In silico and in vitro analysis
Source: Food Chem X. 2026 Jan 16;34:103510. doi: 10.1016/j.fochx.2026.103510 (PMC12861254; doi:10.1016/j.fochx.2026.103510)
Supplement: Supplementary file 1 — Table S1. Peptides identified in the sea cucumber gonad peptide fraction. [file mmc1.docx]

**Table S1** LC-MS/MS analysis of 199 identified gonad peptides from sea cucumber

| Sequence | Length | Mass（Da） | Proteins | Leading razor protein | Score | PSM |
| --- | --- | --- | --- | --- | --- | --- |
| AVSEGTKAVTKYTTSK | 16 | 1669.8887 | A0A2G8KT35;A0A2G8KT32 | A0A2G8KT35 | 266.75 | 2 |
| AGPTGPTGP | 9 | 753.36572 | A0A1I9W676 | A0A1I9W676 | 236.77 | 1 |
| VGAQGERGEAGNTGPQ | 16 | 1526.7073 | A0A1I9W676 | A0A1I9W676 | 229.56 | 1 |
| GPAGPTGPTGPA | 12 | 978.47706 | A0A1I9W676 | A0A1I9W676 | 227.3 | 1 |
| TNWDDMEK | 8 | 1037.4124 | C5NTJ9;A0A2G8JRZ2;A0A2G8KMT7;A0A2G8JBD9;A0A2G8KJ01;A0A2G8JSN3;A0A2G8K0Q5 | C5NTJ9 | 226.55 | 1 |
| GPTGPTGPA | 9 | 753.36572 | A0A1I9W676 | A0A1I9W676 | 224.39 | 1 |
| NPDHPIIE | 8 | 933.4556 | R4HC40;G4XU66;A0A097GVX9;A0A6M2VKR8;A0A2G8JL82 | R4HC40 | 221.25 | 1 |
| AGPTGPTGPAG | 11 | 881.4243 | A0A1I9W676 | A0A1I9W676 | 219.7 | 1 |
| NEATGGKYVPR | 11 | 1190.6044 | A0A2G8KRV2;A0A2G8KRX3;A0A2G8KYX9;A0A2G8KRX5;A0A2G8JR39 | A0A2G8KRV2 | 217.5 | 1 |
| AVSEGTKAVTKYTTS | 15 | 1541.7937 | A0A2G8KT35;A0A2G8KT32 | A0A2G8KT35 | 215.34 | 2 |
| DLKNKANEEVEK | 12 | 1415.7256 | A0A2G8K7R8 | A0A2G8K7R8 | 214.86 | 1 |
| LNDTKGSSGVAIKK | 14 | 1416.7936 | A0A2G8LB13;A0A2G8JRI4;A0A2G8K942;A0A2G8JX95;A0A2G8K918 | A0A2G8LB13 | 213.99 | 2 |
| VGEGMEEGE | 9 | 935.35423 | A0A2G8JN23;A0A2G8K0Q6;A0A2G8K0S7;A0A2G8JYC3;A0A2G8K0R4;A0A0M4ATJ8;A0A2G8JIJ4;A0A2G8LFU4;A0A2G8K0U2;A0A2G8K0R2;A0A2G8LLY4;A0A2G8LPX8;A0A2G8LLX1;A0A2G8JQV5;A0A2G8LFY3;A0A2G8K0P7 | A0A2G8JN23 | 200.66 | 1 |
| SEGTKAVTKYTTS | 13 | 1371.6882 | A0A2G8KT35;A0A2G8KT32 | A0A2G8KT35 | 198.57 | 2 |
| AIADLNDPKGSSGTAIKK | 18 | 1784.9632 | A0A2G8JDJ2;A0A2G8LCY5 | A0A2G8JDJ2 | 189.09 | 3 |
| VTEDEGNEQTDGRP | 14 | 1545.6543 | A0A2G8JTU0 | A0A2G8JTU0 | 188.61 | 1 |
| GVETGVLK | 8 | 801.45962 | A0A2G8JLY2;A0A2G8JDJ2;A0A2G8LCY5 | A0A2G8JLY2 | 186.43 | 1 |
| GAAGAAGAAGSQGNQGER | 18 | 1528.6978 | A0A2G8LKB5 | A0A2G8LKB5 | 186.17 | 1 |
| GATGERGPAGPS | 12 | 1055.4996 | A0A2G8LKB8 | A0A2G8LKB8 | 185.6 | 1 |
| SKPTGPTT | 8 | 787.40758 | A0A2G8LB13;A0A2G8JRI4;A0A2G8K942;A0A2G8JX95;A0A2G8K918 | A0A2G8LB13 | 183.9 | 1 |
| AGPTGPTGPA | 10 | 824.40283 | A0A1I9W676 | A0A1I9W676 | 180.18 | 1 |
| AGDSKKDPPIQAE | 13 | 1354.6729 | A0A2G8JU26;A0A2G8L9Y8 | A0A2G8JU26 | 179.19 | 1 |
| IPLPEEN | 7 | 810.41233 | A0A2G8JKY2 | A0A2G8JKY2 | 178.65 | 1 |
| GGGTGSGMGTL | 11 | 893.39128 | A0A2G8KRV2;A0A2G8KRX3;A0A2G8KYX9;A0A2G8KS26;A0A2G8KRV0 | A0A2G8KRV2 | 168.66 | 1 |
| TPTYGDL | 7 | 765.35448 | A0A2G8KRV2;A0A2G8KRX3;A0A2G8KS26;A0A2G8KRV0;A0A2G8JQW5 | A0A2G8KRV2 | 167.23 | 1 |
| AGDSKKDPPIQAET | 14 | 1455.7205 | A0A2G8JU26;A0A2G8L9Y8 | A0A2G8JU26 | 165.39 | 1 |
| TPTYGDLNH | 9 | 1016.4563 | A0A2G8KRV2;A0A2G8KRX3;A0A2G8KS26;A0A2G8KRV0;A0A2G8JQW5 | A0A2G8KRV2 | 164.53 | 1 |
| ETPIYEQ | 7 | 878.40216 | A0A2G8K7R8 | A0A2G8K7R8 | 163.84 | 1 |
| GQDGEDGRDGAQGPA | 15 | 1428.5866 | A0A1I9W676 | A0A1I9W676 | 163.45 | 1 |
| GPANPDNIPEDGR | 13 | 1350.6164 | A0A2G8LQ00;A0A2G8LQ99 | A0A2G8LQ00 | 162.38 | 1 |
| TGPAGPQGPAGDRGQ | 15 | 1364.6433 | A0A1I9W676 | A0A1I9W676 | 160.22 | 1 |
| DKEGIPPDQQR | 11 | 1281.6313 | A0A2G8LBR5;A0A2G8KUQ6;A0A2G8KDT4;A0A2G8KC12;A0A2G8KC39;A0A2G8KDU5;A0A2G8JG69;A0A2G8JG54;A0A2G8JG56;A0A2G8JG89;A0A2G8JG81 | A0A2G8LBR5 | 159.83 | 1 |
| FGDDISQDNEDNR | 13 | 1523.6124 | E3WHU2 | E3WHU2 | 159.09 | 1 |
| AAGAAGAAGSQGNQGERGRQGAQG | 24 | 2125.9962 | A0A2G8LKB5 | A0A2G8LKB5 | 159.04 | 1 |
| GQRGPAGPTGPTGP | 14 | 1248.6211 | A0A1I9W676 | A0A1I9W676 | 158.94 | 1 |
| GPAGPQGP | 8 | 679.32894 | A0A1I9W676 | A0A1I9W676 | 158.16 | 1 |
| GVATGVLK | 8 | 743.45414 | A0A2G8LB13;A0A2G8JRI4;A0A2G8K942;A0A2G8JX95;A0A2G8K918 | A0A2G8LB13 | 158.12 | 1 |
| APVISAEKAYH | 11 | 1184.619 | A0A2G8JN23;A0A2G8K0Q6;A0A2G8K0S7;A0A2G8K0Q9;A0A2G8JYC3;A0A2G8JIJ4;A0A2G8JQX3;A0A2G8LFU4 | A0A2G8JN23 | 157.96 | 1 |
| IPPGGTEP | 8 | 766.38612 | A0A2G8LH72 | A0A2G8LH72 | 156.93 | 1 |
| ISKEEYDESGPS | 12 | 1339.578 | C5NTJ9;A0A2G8JRZ2 | C5NTJ9 | 156.16 | 1 |
| AVANAQDFSHQ | 11 | 1186.5367 | A0A2G8KWZ0;A0A2G8LM02 | A0A2G8KWZ0 | 155.58 | 1 |
| HKDTPENNPNTP | 12 | 1362.6164 | A0A2G8LRY6 | A0A2G8LRY6 | 150.27 | 1 |
| DNYPRDGKHPTD | 12 | 1413.6273 | A0A2G8LAG4 | A0A2G8LAG4 | 149.96 | 1 |
| ATGPQGPAGQRGPAGPTGPTGPA | 23 | 1998.9872 | A0A1I9W676 | A0A1I9W676 | 149.59 | 1 |
| IDPTGTYHGD | 10 | 1074.4618 | A0A2G8KRV2;A0A2G8KRX3;A0A2G8KYX9;A0A2G8KRX5;A0A2G8JR39 | A0A2G8KRV2 | 149.4 | 1 |
| LPFQR | 5 | 659.37549 | A0A2G8KMC0;A0A2G8LB53;A0A1S6YDW3;A0A2G8K8I7;A0A2G8LN81;A0A2G8K0X6 | A0A2G8KMC0 | 145.19 | 2 |
| SPAPGSEKSRAGQP | 14 | 1367.6793 | A0A2G8KGP3 | A0A2G8KGP3 | 143.97 | 2 |
| ISGTSMATPH | 10 | 1000.4648 | W6FIM7;Q1WDP1;A0A2G8K8I3;A0A2G8K9E4 | W6FIM7 | 143.85 | 1 |
| REEDIPPGADGR | 12 | 1310.6215 | A0A2G8L9T0 | A0A2G8L9T0 | 142.19 | 2 |
| FDDNIGDHE | 9 | 1060.4098 | A0A2G8KWZ0;A0A2G8LM02 | A0A2G8KWZ0 | 141.39 | 1 |
| NRKSTITSREVQT | 13 | 1518.8114 | A0A2G8KT35;A0A2G8KT32 | A0A2G8KT35 | 141.37 | 2 |
| IDGVEGRQGPA | 11 | 1097.5465 | A0A2G8LKB5 | A0A2G8LKB5 | 140.83 | 1 |
| GQRGPAGPTGPTGPAGA | 17 | 1447.7168 | A0A1I9W676 | A0A1I9W676 | 140.14 | 1 |
| GQRGPAGPTGP | 11 | 993.49919 | A0A1I9W676 | A0A1I9W676 | 138.26 | 1 |
| EGPQGPNGERGPTGD | 15 | 1466.6386 | A0A3G1ZIU9;A0A2G8LQN2 | A0A3G1ZIU9 | 138.24 | 1 |
| GQRGPAGPTGPTGPAG | 16 | 1376.6797 | A0A1I9W676 | A0A1I9W676 | 137.55 | 1 |
| AQGERGEAGNTGPQ | 14 | 1370.6175 | A0A1I9W676 | A0A1I9W676 | 136.81 | 1 |
| GYRGSQGERGPYG | 13 | 1382.6327 | A0A2G8LKB5 | A0A2G8LKB5 | 136.5 | 1 |
| LNDTKGSSGVAIK | 13 | 1288.6987 | A0A2G8LB13;A0A2G8JRI4;A0A2G8K942;A0A2G8JX95;A0A2G8K918 | A0A2G8LB13 | 135.26 | 1 |
| TGVDNPGHPF | 10 | 1039.4723 | A0A2G8LAG4 | A0A2G8LAG4 | 135 | 1 |
| HGPTAPFEGNKEA | 13 | 1353.6313 | A0A2G8LFZ4;A0A2G8JEV0 | A0A2G8LFZ4 | 134.57 | 1 |
| PGGDLAKVQR | 10 | 1039.5774 | A0A2G8JN23;A0A2G8K0Q6;A0A2G8K0S7;A0A2G8K0R1;A0A2G8JYC3;A0A2G8K0R4;A0A0M4ATJ8;A0A2G8K0R2;A0A2G8LPX8;A0A2G8LLX1;A0A2G8JQV5 | A0A2G8JN23 | 133.32 | 1 |
| VPGTGTDGSGLGHES | 15 | 1369.611 | A0A2G8KMF0 | A0A2G8KMF0 | 132.39 | 1 |
| QVHPDTGISSRAMS | 14 | 1484.7042 | A0A2G8KT35;A0A2G8KT32 | A0A2G8KT35 | 131.82 | 2 |
| YVGDEAQSKRG | 11 | 1208.5786 | C5NTJ9;A0A2G8JRZ2;A0A2G8KMT7;A0A2G8JBD9;A0A2G8KJ01;A0A2G8JSN3;A0A2G8K0Q5 | C5NTJ9 | 131.82 | 2 |
| GATGERGPSGP | 11 | 984.46247 | A0A1I9W676 | A0A1I9W676 | 131.52 | 1 |
| VPGTGTDGSGLGHESG | 16 | 1426.6325 | A0A2G8KMF0 | A0A2G8KMF0 | 131.46 | 1 |
| AVSEGTKAVTKY | 12 | 1252.6663 | A0A2G8KT35;A0A2G8KT32 | A0A2G8KT35 | 131.42 | 1 |
| KQVHPDTGISSRAMS | 15 | 1612.7991 | A0A2G8KT35;A0A2G8KT32 | A0A2G8KT35 | 130.72 | 2 |
| LPVLD | 5 | 555.32681 | A0A2G8JUA3 | A0A2G8JUA3 | 129.54 | 1 |
| SYVGDEAQSKRG | 12 | 1295.6106 | C5NTJ9;A0A2G8JRZ2;A0A2G8KMT7;A0A2G8JBD9;A0A2G8KJ01;A0A2G8JSN3;A0A2G8K0Q5 | C5NTJ9 | 128.52 | 1 |
| QRGPAGPTGPTGPA | 14 | 1262.6367 | A0A1I9W676 | A0A1I9W676 | 128.27 | 1 |
| ATGPQGQQGSRGERGPEGQQG | 21 | 2080.9635 | A0A1I9W676 | A0A1I9W676 | 127.32 | 1 |
| GQRGPAGPTGPT | 12 | 1094.5469 | A0A1I9W676 | A0A1I9W676 | 125.97 | 1 |
| KDTGSPIRIP | 10 | 1082.6084 | A0A2G8JRT9 | A0A2G8JRT9 | 125.75 | 1 |
| FDGDVGDQG | 9 | 908.35119 | A0A2G8LKB5 | A0A2G8LKB5 | 125.53 | 1 |
| EEGEF | 5 | 609.22822 | A0A2G8JN23;A0A2G8K0Q6;A0A2G8K0S7;A0A2G8JYC3;A0A2G8K0R4;A0A0M4ATJ8;A0A2G8JIJ4;A0A2G8LFU4;A0A2G8K0U2;A0A2G8K0R2;A0A2G8LLY4;A0A2G8LPX8;A0A2G8LLX1;A0A2G8JQV5;A0A2G8K0P7;A0A2G8JDR7;A0A2G8JN38;A0A2G8LQ66;A0A2G8KD09;A0A2G8L2J3;A0A2G8K0U6;A0A2G8LH39;A0A2G8LH66;A0A2G8JKJ9;A0A2G8JQ73;A0A2G8KRL6;A0A2G8LHA5;A0A2G8KRV2;A0A2G8KRX3;A0A2G8KS26;A0A2G8JQW5;A0A2G8KRV7 | A0A2G8KRV2 | 124.87 | 1 |
| GQRGPAGPTGPTGPAGATGERGPSGPQ | 27 | 2414.1687 | A0A1I9W676 | A0A1I9W676 | 124.51 | 1 |
| AVSEGTKAVTK | 11 | 1089.603 | A0A2G8KT35;A0A2G8KT32 | A0A2G8KT35 | 123.96 | 2 |
| VPFPR | 5 | 614.35403 | A0A2G8KRV2;A0A2G8KRX3;A0A2G8KYX9;A0A2G8KS26;A0A2G8KRV0;A0A2G8JQW5;A0A2G8KRV7;A0A2G8JDG4;A0A2G8K9U2 | A0A2G8KRV2 | 123.86 | 1 |
| ATGPQGPAGQRGPAGPTGPTGPAG | 24 | 2056.0086 | A0A1I9W676 | A0A1I9W676 | 122.93 | 1 |
| HSLGGGTGSGMGT | 13 | 1117.4822 | A0A2G8KRV2;A0A2G8KRX3;A0A2G8KYX9;A0A2G8KS26;A0A2G8KRV0 | A0A2G8KRV2 | 122.28 | 1 |
| IGGSSNNDSGKVD | 13 | 1248.5582 | A0A2G8LNP3 | A0A2G8LNP3 | 121.96 | 1 |
| DKEGIPPDQQRL | 12 | 1394.7154 | A0A2G8LBR5;A0A2G8KUQ6;A0A2G8KDT4;A0A2G8KC12;A0A2G8KC39;A0A2G8KDU5;A0A2G8JG69;A0A2G8JG54;A0A2G8JG56;A0A2G8JG89 | A0A2G8LBR5 | 121.6 | 2 |
| GSTGPAGPQGPAGDR | 15 | 1323.6167 | A0A1I9W676 | A0A1I9W676 | 121.02 | 1 |
| LPVNE | 5 | 570.30133 | E3WHU2;A0A2G8JZP6;A0A2G8JGB7;A0A2G8LP07;A0A2G8JSY8;A0A2G8JFX5;A0A2G8JHV2;A0A2G8JL41 | E3WHU2 | 120.06 | 1 |
| SKLEF | 5 | 622.33263 | A0A2G8JN23;A0A2G8K0Q6;A0A2G8K0S7;A0A2G8K0R1;A0A2G8K0Q9;A0A2G8JYC3;A0A2G8K0R4;A0A2G8JIJ4;A0A2G8JQX3;A0A2G8K0R2;A0A2G8LLY4;A0A2G8JWX4;A0A2G8KQS3;A0A2G8JEJ7;A0A2G8KTS4 | A0A2G8JN23 | 120.06 | 2 |
| APLNP | 5 | 510.2802 | C5NTJ9;A0A2G8JRZ2;A0A2G8KMT7;A0A2G8KMS5;A0A8G1M2U6;A0A2G8LMH4;A0A2G8L5J3;A0A2G8KSZ9;A0A2G8LCM7;A0A2G8KDB9;A0A2G8LAD7;A0A2G8K9B4;A0A2G8JJD8;A0A2G8JHC0;A0A2G8L932;A0A2G8JH86;A0A2G8K937;A0A2G8LEF4 | C5NTJ9 | 120 | 1 |
| GPFGQ | 5 | 504.23325 | A0A2G8KRV2;A0A2G8KRX3;A0A2G8KYX9;A0A2G8KS26;A0A2G8KRV0;A0A2G8KRX5;A0A2G8KRV7;A0A2G8JR39 | A0A2G8KRV2 | 120 | 1 |
| KQVHPDTGISSRAM | 14 | 1525.7671 | A0A2G8KT35;A0A2G8KT32 | A0A2G8KT35 | 119.45 | 2 |
| AVSEGTKAVTKYT | 13 | 1353.714 | A0A2G8KT35;A0A2G8KT32 | A0A2G8KT35 | 119.03 | 1 |
| LPGEL | 5 | 527.29551 | A0A2G8KT35;A0A2G8KT32;A0A2G8LJV4;A0A2G8KQV0;A0A2G8LCG8;A0A2G8KKV2 | A0A2G8KT35 | 116.54 | 1 |
| TGEGMDEME | 9 | 997.33686 | A0A2G8KRV2;A0A2G8KRX3;A0A2G8KYX9;A0A2G8KS26;A0A2G8KRV0;A0A2G8JQW5;A0A2G8KRV7 | A0A2G8KRV2 | 116.54 | 1 |
| GVATGVLKQ | 9 | 871.51272 | A0A2G8LB13;A0A2G8JRI4;A0A2G8K942;A0A2G8JX95;A0A2G8K918 | A0A2G8LB13 | 116.3 | 1 |
| SEGTKAVTKYTTSK | 14 | 1499.7831 | A0A2G8KT35;A0A2G8KT32 | A0A2G8KT35 | 114.69 | 1 |
| AAEASRLAHY | 10 | 1087.5411 | A0A2G8KT35;A0A2G8KT32 | A0A2G8KT35 | 113.74 | 1 |
| SKPTGPTTL | 9 | 900.49165 | A0A2G8LB13;A0A2G8JRI4;A0A2G8K942;A0A2G8JX95;A0A2G8K918 | A0A2G8LB13 | 113.5 | 1 |
| YVPPAEDSEEQR | 12 | 1418.6314 | A0A2G8KGP3 | A0A2G8KGP3 | 112.75 | 1 |
| NPWGQ | 5 | 600.26561 | A0A2G8KKC3 | A0A2G8KKC3 | 112.58 | 1 |
| VPVVN | 5 | 526.3115 | A0A2G8JTU0;A0A2G8JRH1;A0A2G8LE08;A0A2G8JQF7;A0A1W5LDR6;A0A2G8K9S1 | A0A2G8JTU0 | 112.31 | 1 |
| STGPAGPQGPAGDRGQ | 16 | 1451.6753 | A0A1I9W676 | A0A1I9W676 | 111.96 | 1 |
| GHQGPRGDQGEDGADGRDGA | 20 | 1950.8165 | A0A2G8LKB5 | A0A2G8LKB5 | 111.95 | 2 |
| AVSEGTKAVTKYTT | 14 | 1454.7617 | A0A2G8KT35;A0A2G8KT32 | A0A2G8KT35 | 111.94 | 1 |
| IGGIGTVPVGR | 11 | 1024.6029 | A0A2G8JU26;A0A2G8L9Y8 | A0A2G8JU26 | 110.87 | 1 |
| VAGDSKKDPPIQAET | 15 | 1554.789 | A0A2G8JU26;A0A2G8L9Y8 | A0A2G8JU26 | 110.08 | 1 |
| RGTPGGPDKKGEAGAGSTDN | 20 | 1870.8769 | A0A2G8KKS8;A0A2G8JEL8 | A0A2G8KKS8 | 109.66 | 1 |
| ATGERGPSGPQ | 11 | 1055.4996 | A0A1I9W676 | A0A1I9W676 | 109.02 | 1 |
| GPAGPQGEVGDR | 12 | 1138.5367 | A0A1I9W676 | A0A1I9W676 | 108.43 | 1 |
| SVGESGSRGPAGA | 13 | 1130.5316 | A0A2G8LKB5 | A0A2G8LKB5 | 107.79 | 1 |
| REEDIPPGADGRR | 13 | 1466.7226 | A0A2G8L9T0 | A0A2G8L9T0 | 106.32 | 2 |
| YNEATGGKYVPR | 12 | 1353.6677 | A0A2G8KRV2;A0A2G8KRX3;A0A2G8KYX9;A0A2G8KRX5;A0A2G8JR39 | A0A2G8KRV2 | 106.29 | 2 |
| SETGAGKHVPRA | 12 | 1208.6262 | A0A2G8JN23;A0A2G8K0Q6;A0A2G8K0S7;A0A2G8K0R1;A0A2G8K0Q9 | A0A2G8JN23 | 105.4 | 1 |
| VTYAP | 5 | 549.27986 | A0A2G8JN23;A0A0M4ATJ8;A0A2G8JQX3;A0A2G8LFU4;A0A2G8K0U2 | A0A2G8JN23 | 105.36 | 1 |
| SEGPF | 5 | 535.22783 | A0A2G8K7R8;A0A2G8LKH2;A0A2G8KXX8;A0A2G8LR75 | A0A2G8K7R8 | 104.62 | 1 |
| MPPPP | 5 | 537.2621 | A0A2G8LAJ9;A0A2G8K445;A0A2G8KTY1;A0A2G8KFI6;A0A2G8JUN5;A0A2G8KTW3;A0A2G8JS09;A0A2G8JYC4;A0A2G8LGP3;A0A2G8KQX3;A0A2G8L9W2;A0A2G8JZX7;A0A2G8L5Q0;A0A2G8LFY0;A0A2G8JZH9;A0A2G8JDW3;A0A2G8LB29;A0A2G8K151;A0A2G8JBS4 | A0A2G8LAJ9 | 104.08 | 1 |
| PKGSSGTAIKK | 11 | 1072.6241 | A0A2G8JDJ2;A0A2G8LCY5 | A0A2G8JDJ2 | 103.91 | 1 |
| REEDIPPGADGRRE | 14 | 1595.7652 | A0A2G8L9T0 | A0A2G8L9T0 | 103.3 | 1 |
| GPTAPFEGNKEA | 12 | 1216.5724 | A0A2G8LFZ4;A0A2G8JEV0 | A0A2G8LFZ4 | 102.06 | 1 |
| IDPTGTYHGDSD | 12 | 1276.5208 | A0A2G8KRV2;A0A2G8KRX3;A0A2G8KYX9;A0A2G8KRX5;A0A2G8JR39 | A0A2G8KRV2 | 101.64 | 1 |
| KQVHPDTGISSR | 12 | 1323.6895 | A0A2G8KT35;A0A2G8KT32 | A0A2G8KT35 | 101.39 | 1 |
| ATGPQGPAGQRGPA | 14 | 1263.632 | A0A1I9W676 | A0A1I9W676 | 99.864 | 1 |
| ALKRGVETGVLK | 12 | 1269.7769 | A0A2G8JLY2;A0A2G8JDJ2;A0A2G8LCY5 | A0A2G8JLY2 | 99.788 | 2 |
| FPGQL | 5 | 560.29585 | A0A2G8KRV2;A0A2G8KRX3;A0A2G8KYX9;A0A2G8KS26;A0A2G8KRV0;A0A2G8JQW5;A0A2G8KRV7;A0A2G8LAW6;A0A2G8KFM9;A0A2G8KAU0 | A0A2G8KRV2 | 99.626 | 1 |
| GPVQY | 5 | 562.27511 | A0A2G8LKB8;A0A2G8L3B4;A0A2G8KJU7 | A0A2G8LKB8 | 99.605 | 1 |
| PERKY | 5 | 691.36532 | C5NTJ9;A0A2G8JRZ2;A0A2G8KMT7;A0A2G8KMS5;A0A8G1M2U6;A0A2G8JBD5;A0A2G8KJ01;A0A2G8JSN3 | C5NTJ9 | 97.926 | 1 |
| APMNP | 5 | 528.23662 | A0A2G8KJ01;A0A2G8JSN3;A0A2G8K0Q5;A0A2G8K6W8;A0A2G8L0H8 | A0A2G8KJ01 | 97.866 | 2 |
| SDYNIQKESTLH | 12 | 1433.6787 | A0A2G8LBR5;A0A2G8KUQ6;A0A2G8KDT4;A0A2G8KC12;A0A2G8KC39;A0A2G8KDU5 | A0A2G8LBR5 | 96.342 | 1 |
| EHPVL | 5 | 593.31731 | C5NTJ9;A0A2G8JRZ2;A0A2G8KMT7;A0A2G8KMS5;A0A8G1M2U6;A0A2G8L5J3;A0A2G8LCI1;A0A2G8KJ01;A0A2G8JSN3 | C5NTJ9 | 94.533 | 1 |
| ATGPQGQQGSRGERGPEGQQGQAG | 24 | 2337.0806 | A0A1I9W676 | A0A1I9W676 | 94.359 | 1 |
| IAALNDSKGSSGSAIKK | 17 | 1645.8999 | A0A2G8JLY2 | A0A2G8JLY2 | 94.203 | 1 |
| IDERGPIPTDRR | 12 | 1423.7532 | A0A2G8JRT9 | A0A2G8JRT9 | 93.494 | 1 |
| PSNLGTGLR | 9 | 913.49813 | A0A2G8LAG4;A0A2G8LAS7;A0A2G8LAG8;A0A2G8JGI3 | A0A2G8LAG4 | 93.374 | 1 |
| IADLNDPKGSSGTAIKK | 17 | 1713.9261 | A0A2G8JDJ2;A0A2G8LCY5 | A0A2G8JDJ2 | 93.152 | 1 |
| LELPEDEEEKKK | 12 | 1485.7563 | R4HC40;G4XU66;A0A097GVX9;A0A6M2VKR8;A0A2G8JL82;D0V3X9 | R4HC40 | 91.961 | 1 |
| GERGEAGNTGPQ | 12 | 1171.5218 | A0A1I9W676 | A0A1I9W676 | 90.827 | 1 |
| VAGTP | 5 | 443.238 | A0A3G1ZIU9;A0A2G8JPE5 | A0A3G1ZIU9 | 90 | 1 |
| VVPGGDLAKVQR | 12 | 1237.7143 | A0A2G8JN23;A0A2G8K0Q6;A0A2G8K0S7;A0A2G8K0R1;A0A2G8JYC3;A0A2G8K0R4;A0A0M4ATJ8;A0A2G8K0R2;A0A2G8LPX8;A0A2G8LLX1 | A0A2G8JN23 | 89.403 | 1 |
| LPGPP | 5 | 479.27438 | A0A1I9W676;A0A2G8LCI5;A0A2G8JJU8;A0A2G8LI61;A0A2G8JW32;A0A2G8LQX1;A0A2G8JL05;A0A2G8LR12 | A0A1I9W676 | 88.98 | 1 |
| GEVGDRGNPGPA | 12 | 1124.521 | A0A1I9W676 | A0A1I9W676 | 88.496 | 1 |
| GDSKKDPPIQAET | 13 | 1384.6834 | A0A2G8JU26;A0A2G8L9Y8 | A0A2G8JU26 | 88.187 | 1 |
| YNEATGGKYVPRA | 13 | 1424.7048 | A0A2G8KRV2;A0A2G8KRX3;A0A2G8KYX9;A0A2G8KRX5;A0A2G8JR39 | A0A2G8KRV2 | 88.134 | 1 |
| NEATGGKYVPRA | 12 | 1261.6415 | A0A2G8KRV2;A0A2G8KRX3;A0A2G8KYX9;A0A2G8KRX5;A0A2G8JR39 | A0A2G8KRV2 | 86.898 | 1 |
| GATGERGPSGPQ | 12 | 1112.521 | A0A1I9W676 | A0A1I9W676 | 86.879 | 1 |
| LPLQD | 5 | 584.31698 | A0A2G8JU26;A0A2G8L9Y8;A0A2G8KNN3;A0A2G8L2W7;A0A2G8JV31 | A0A2G8JU26 | 86.344 | 1 |
| RPKDPADNEAG | 11 | 1168.5473 | E3WHU2 | E3WHU2 | 85.731 | 1 |
| SILTTHTTLEHS | 12 | 1338.6779 | A0A2G8JN23;A0A2G8K0Q6;A0A2G8K0S7;A0A2G8K0R1;A0A2G8K0R4 | A0A2G8JN23 | 85.676 | 1 |
| GQQGSRGERGPEGQQGQAG | 19 | 1882.863 | A0A1I9W676 | A0A1I9W676 | 85.362 | 1 |
| SGVERSSVFINK | 12 | 1321.699 | A0A2G8JLY2 | A0A2G8JLY2 | 85.355 | 1 |
| VGESGSRGPAGA | 12 | 1043.4996 | A0A2G8LKB5 | A0A2G8LKB5 | 83.647 | 1 |
| MESAGIHETTYN | 12 | 1351.5714 | C5NTJ9;A0A2G8JRZ2;A0A2G8JBD5;A0A2G8KJ01;A0A2G8JSN3 | C5NTJ9 | 82.749 | 1 |
| WAKGHYTEGAE | 11 | 1247.5571 | A0A2G8KRV2;A0A2G8KRX3;A0A2G8KYX9;A0A2G8KS26;A0A2G8KRV0;A0A2G8KRX5;A0A2G8KRV7 | A0A2G8KRV2 | 81.972 | 1 |
| RKSTITSREVQT | 12 | 1404.7685 | A0A2G8KT35;A0A2G8KT32 | A0A2G8KT35 | 81.565 | 1 |
| YRPGT | 5 | 592.29691 | A0A2G8KMC0;A0A2G8LB53;A0A1S6YDW3 | A0A2G8KMC0 | 80.317 | 1 |
| QVHPDTGISSRA | 12 | 1266.6317 | A0A2G8KT35;A0A2G8KT32 | A0A2G8KT35 | 77.662 | 1 |
| VAGDSKKDPPIQA | 13 | 1324.6987 | A0A2G8JU26;A0A2G8L9Y8 | A0A2G8JU26 | 77.288 | 1 |
| APLLL | 5 | 525.35263 | A0A2G8KKS8;A0A2G8K958;A0A2G8L3H9;A0A2G8JI08;A0A2G8KLQ3;A0A2G8KN52;A0A2G8L3K6;A0A2G8JEY4;A0A2G8JRJ7;A0A2G8JIW4;A0A2G8KU50 | A0A2G8KKS8 | 76.733 | 1 |
| VGYNP | 5 | 548.25946 | A0A2G8JU26;A0A2G8L9Y8 | A0A2G8JU26 | 76.287 | 1 |
| YEPTP | 5 | 605.26969 | E3WHU2;A0A2G8JZP6;A0A2G8LL60;A0A2G8JCR5;A0A2G8K5N6;A0A2G8L302 | E3WHU2 | 76.053 | 1 |
| DLVVK | 5 | 572.35336 | A0A2G8LB13;A0A2G8JRI4;A0A2G8K942;A0A2G8JX95;A0A2G8K918;A0A2G8LNI6;A0A2G8JLG1;A0A2G8JIF0;A0A2G8KHE9;A0A2G8JKD4;A0A2G8KAA0;A0A2G8L9R0;A0A2G8KA91;A0A2G8LJE6;A0A2G8JLY2 | A0A2G8LB13 | 74.849 | 2 |
| DMKVF | 5 | 638.30978 | A0A2G8K7R8;A0A2G8JE68;A0A2G8LAH5;A0A2G8JIT3 | A0A2G8K7R8 | 74.849 | 1 |
| GNQGPRGGPGETGK | 14 | 1310.6327 | A0A2G8LKB5 | A0A2G8LKB5 | 74.76 | 1 |
| SAGIHETTYNS | 11 | 1178.5204 | C5NTJ9;A0A2G8JRZ2;A0A2G8JBD5;A0A2G8KJ01;A0A2G8JSN3 | C5NTJ9 | 74.301 | 1 |
| AATAAPQPGGPQ | 12 | 1064.5251 | A0A2G8LAJ9 | A0A2G8LAJ9 | 74.267 | 1 |
| LNDPKGSSGTAIKK | 14 | 1414.778 | A0A2G8JDJ2;A0A2G8LCY5 | A0A2G8JDJ2 | 73.927 | 1 |
| QPDGQMPSDKT | 11 | 1202.5238 | A0A2G8JN23;A0A2G8K0Q6;A0A2G8K0S7;A0A2G8K0R1;A0A2G8K0Q9;A0A0M4ATJ8;A0A2G8LLW7 | A0A2G8JN23 | 73.885 | 1 |
| FSETGAGKHVPR | 12 | 1284.6575 | A0A2G8JN23;A0A2G8K0Q6;A0A2G8K0S7;A0A2G8K0R1;A0A2G8K0Q9 | A0A2G8JN23 | 73.848 | 1 |
| GPDASEGDRSRGGGPGR | 17 | 1626.7459 | A0A2G8KJU7 | A0A2G8KJU7 | 73.802 | 1 |
| PGHPF | 5 | 553.26488 | A0A2G8LAG4 | A0A2G8LAG4 | 73.666 | 1 |
| LGGPL | 5 | 455.27438 | A0A2G8K8A9;A0A2G8JGL5 | A0A2G8K8A9 | 73.666 | 1 |
| GPLGP | 5 | 439.24308 | A0A1I9W676;A0A2G8JXZ2;A0A2G8L1E9;A0A2G8JL57 | A0A1I9W676 | 73.666 | 1 |
| SETGAGKHVPR | 11 | 1137.5891 | A0A2G8JN23;A0A2G8K0Q6;A0A2G8K0S7;A0A2G8K0R1;A0A2G8K0Q9 | A0A2G8JN23 | 73.499 | 1 |
| LPLKL | 5 | 582.41048 | A0A2G8K8A9;A0A2G8LG37;A0A2G8L771;A0A2G8KA51;A0A2G8LLS2;A0A2G8KIC2;A0A2G8KWY8;A0A2G8KG43;A0A2G8JS13 | A0A2G8K8A9 | 73.289 | 2 |
| GPMGP | 5 | 457.1995 | A0A3G1ZIU9;A0A2G8KE87;A0A2G8L2U7;A0A2G8LAJ5;A0A2G8L2V7;A0A2G8K7R0;A0A2G8L2V6;A0A2G8LBW2;A0A2G8KN86;A0A2G8L2W8;A0A2G8KWA5;A0A2G8KEA4;A0A2G8JCM0;A0A2G8LJG5;A0A2G8LI68;A0A2G8JH64 | A0A3G1ZIU9 | 72.087 | 1 |
| GPVGL | 5 | 441.25873 | A0A1I9W676;A0A2G8LGT0;A0A2G8KUP4;A0A2G8KUH5;A0A2G8KBY4;A0A2G8JQQ4;A0A2G8L6W1;A0A146AXJ2;A0A2G8LKB5 | A0A1I9W676 | 72.087 | 1 |
| VPLGM | 5 | 515.27775 | A0A2G8K8A9;A0A2G8K250 | A0A2G8K8A9 | 72.087 | 1 |
| KQVHPDTGISSRA | 13 | 1394.7266 | A0A2G8KT35;A0A2G8KT32 | A0A2G8KT35 | 71.879 | 1 |
| EPGTM | 5 | 533.21555 | A0A2G8KRV2;A0A2G8KRX3;A0A2G8KYX9;A0A2G8KRV0;A0A2G8KRX5;A0A2G8JR39 | A0A2G8KRV2 | 71.475 | 1 |
| VHPDTGISSRAM | 12 | 1269.6136 | A0A2G8KT35;A0A2G8KT32 | A0A2G8KT35 | 71.349 | 1 |
| GPDGQAGERGPRGPQ | 15 | 1477.7022 | A0A2G8LKB5 | A0A2G8LKB5 | 71.279 | 1 |
| NKANEEVEKMK | 11 | 1318.6551 | A0A2G8K7R8 | A0A2G8K7R8 | 71.085 | 1 |
| GPRGF | 5 | 532.27578 | A0A1I9W676;A0A2G8LDT5;A0A2G8LAM5;A0A2G8K9L0;A0A2G8LNE5;A0A2I4JGM2;A0A2G8JV51;A0A2G8KPD1;A0A2G8JD78;A0A2G8JDP7 | A0A1I9W676 | 71.054 | 1 |
| ERLLK | 5 | 657.41736 | A0A2G8LAG4;A0A2G8JI79;A0A2G8KLE2;A0A2G8L8Y8;A0A2G8KEY4;A0A2G8JQU4;A0A2G8L8M6;A0A7T1TTB6;A0A2G8KVJ4;A0A2G8K7Z7;A0A2G8KZU6;A0A2G8LRY4;A0A2G8LP48;A0A2G8JQR2;A0A2G8KXX9;A0A2G8KJQ1;A0A2G8LN27;A0A2G8LEG7;A0A2G8JB60;A0A6M2VJT4;A0A2G8K9M7;A0A2G8JKQ2;A0A2G8LBF6;A0A2G8LJG9 | A0A2G8LAG4 | 69.39 | 1 |
| YAPVISAEKAYH | 12 | 1347.6823 | A0A2G8JN23;A0A2G8K0Q6;A0A2G8K0S7;A0A2G8K0Q9;A0A2G8JYC3;A0A2G8JIJ4;A0A2G8JQX3;A0A2G8LFU4 | A0A2G8JN23 | 69.276 | 1 |
| FDGPEGPRGPPGSEGRQG | 18 | 1795.8238 | A0A1I9W676 | A0A1I9W676 | 69.188 | 1 |
| GDRGF | 5 | 550.24996 | A0A3G1ZIU9;A0A2G8L191;A0A2G8LI18 | A0A3G1ZIU9 | 67.76 | 1 |
| VTVLE | 5 | 559.32173 | A0A2G8JJL6 | A0A2G8JJL6 | 67.327 | 1 |
| PLTGP | 5 | 483.2693 | A0A2G8K7R8;A0A2G8KQ67 | A0A2G8K7R8 | 66.538 | 1 |
| LELPEDEEEKKKREE | 15 | 1899.9426 | R4HC40;G4XU66;A0A097GVX9;A0A6M2VKR8;A0A2G8JL82 | R4HC40 | 64.52 | 1 |
| TYRYL | 5 | 714.37008 | A0A2G8K7R8;A0A2G8JFE3;A0A2G8KWH9;A0A2G8JT85;A0A2G8JIT7;A0A2G8JJC4 | A0A2G8K7R8 | 64.081 | 1 |
| TGPDGQAGERGPR | 13 | 1296.6171 | A0A2G8LKB5 | A0A2G8LKB5 | 63.662 | 1 |
| SPVLD | 5 | 529.27478 | A0A2G8JU26;A0A2G8L9Y8;A0A2G8JYA9;A0A2G8JSJ9;S6BN65;A0A2G8L870;A0A2G8KEA0;A0A2G8LEC8;A0A2G8JT78;A0A2G8KMJ9;A0A2G8K110;A0A2G8JXL2 | A0A2G8JU26 | 63.581 | 1 |
| DFKTE | 5 | 638.29116 | A0A2G8KMC0;A0A2G8LB53;A0A1S6YDW3;A0A2G8KP28;A0A2G8KGA7;A0A2G8LNX0;A0A2G8L343 | A0A2G8KMC0 | 63.443 | 1 |
| LNDSKGSSGSAIKK | 14 | 1390.7416 | A0A2G8JLY2 | A0A2G8JLY2 | 62.042 | 1 |
| LPVQP | 5 | 552.32715 | A0A2G8KDD2;A0A2G8JJD0 | A0A2G8KDD2 | 61.26 | 1 |
| VGNLP | 5 | 498.2802 | A0A2G8KJU7 | A0A2G8KJU7 | 61.26 | 1 |
| VGSLL | 5 | 487.3006 | A0A2G8K8A9;A0A2G8JCN3;A0A2G8KMQ9;A0A2G8L7X7;A0A2G8L1T3;A0A2G8KB84;A0A2G8LPT8;A0A2G8KGG6;A0A2G8KIB5;A0A2G8L1K2;A0A2G8LRP0;A0A2G8JLB7;A0A2G8JR63;A0A2G8LHB9;A0A2G8LK16;A0A2G8JJ76;A0A2G8JDC1;A0A2G8LQY5;A0A2G8KMC7;A0A2G8LEA6;A0A2G8KNK7;A0A2G8KNK6 | A0A2G8K8A9 | 60.874 | 1 |
| VPGGDLAKVQR | 11 | 1138.6459 | A0A2G8JN23;A0A2G8K0Q6;A0A2G8K0S7;A0A2G8K0R1;A0A2G8JYC3;A0A2G8K0R4;A0A0M4ATJ8;A0A2G8K0R2;A0A2G8LPX8;A0A2G8LLX1;A0A2G8JQV5 | A0A2G8JN23 | 60.255 | 1 |
| TQQMF | 5 | 653.2843 | A0A2G8KRV2;A0A2G8KRX3;A0A2G8KYX9;A0A2G8KS26;A0A2G8KRV0;A0A2G8JQW5;A0A2G8KNV3;A0A2G8LAE6 | A0A2G8KRV2 | 59.539 | 1 |
| VAAVF | 5 | 505.29003 | A0A2G8KRV2;A0A2G8LF82;A0A2G8K9G2;A0A2G8KAM9;A0A2G8KB76;A0A2G8KDM5;A0A2G8L1F7 | A0A2G8KRV2 | 59.298 | 1 |
